# Structural Color Inkjet Printing With Mie‐Resonant Silicon Nanoparticles

**DOI:** 10.1002/adma.202523036

**Published:** 2026-04-03

**Authors:** Hiroto Yamana, Haruki Tanaka, Hiroshi Sugimoto, Minoru Fujii

**Affiliations:** ^1^ Department of Electrical and Electronic Engineering Graduate School of Engineering Kobe University Kobe Japan

**Keywords:** color asymmetry, inkjet printing, Mie resonance, silicon nanoparticle, structural color

## Abstract

Inkjet printing of silicon nanoparticle (Si NP) inks and their inherent color asymmetry in reflection and transmission is demonstrated. The optical response of Si NP‐dispersed films was initially analyzed using Monte Carlo simulations, which elucidated the physical origin of the asymmetric color appearance. Guided by these theoretical insights, water‐based Si NP inks incorporating an acrylic resin were formulated and applied to inkjet printing. The printed films exhibited vivid structural colors with pronounced differences between their reflective and transmissive hues. Furthermore, multicolor patterns with tunable optical asymmetry were produced by employing Si NPs of different diameters. These results highlight the potential of Si NPs as scalable structural‐color pigments for multicolor dichroic decorations in applications such as art, anti‐counterfeiting, and semitransparent smart windows.

## Graphical abstract

Water‐based inks incorporating Mie‐resonant silicon nanoparticles were developed, and their application to structural color inkjet printing was demonstrated. By controlling the nanoparticle size and concentration, coloration covering a wide color gamut was achieved. The printed films exhibit pronounced reflection/transmission color asymmetry arising from Mie resonances, enabling multicolor dichroic designs, offering functionalities unattainable with conventional pigments.

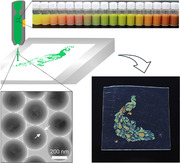

## 1 Introduction

Structural color, known for its superior stability and durability compared to traditional organic pigments, offers a promising approach to material coloration. Originating from interference, diffraction, and scattering of light by nanostructures, structural color does not fade unless the underlying structure is physically altered. The most widely studied mechanism involves the interference or diffraction of light within periodically aligned polymer or transparent oxide nanostructures. While this method enables broad color tunability, it often exhibits strong iridescence [1, 2, 3, 4, 5], which limits its practical applications. Moreover, due to the relatively low refractive index of these materials, coating with a protective layer tends to significantly diminish the perceived color. An alternative approach utilizes light absorption via localized surface plasmon resonances (LSPRs) of noble metal nanoparticles (NPs) [6, 7, 8, 9, 10, 11]. This method does not require periodic ordering, as the color originates from light absorption by individual particles, resulting in non‐iridescent coloration. The LSPR wavelength depends predominantly on the aspect ratio of the nanostructure, allowing color control through structural design. Similarly, high‐refractive‐index dielectric NPs exhibiting low‐order Mie resonances in the visible range can produce non‐iridescent structural colors [12, 13, 14, 15, 16, 17, 18, 19, 20]. Unlike LSPR‐based coloration, which relies on absorption, Mie resonance‐based coloration arises from strong light scattering [21]. A key advantage of this method is the ability to achieve a wide color gamut simply by tuning the particle size, as the Mie resonance wavelength is highly sensitive to size variations [22, 23]. Furthermore, Mie resonances exhibit a notable feature: pronounced asymmetry between forward and backward scattering. Forward scattering arises from in‐phase far‐field interference between the magnetic dipole (MD) and electric dipole (ED) modes (first Kerker condition), whereas backward scattering arises from the out‐of‐phase interference (second Kerker condition) [24, 25, 26]. This effect manifests as a strong reflection/transmission asymmetry in films composed of high‐refractive index nanostructures [27, 28, 29, 30].

The objective of this work is to develop a process for structural coloration of substances, including three‐dimensional objects, using inks composed of Mie‐resonant NPs. To this end, we employed spherical crystalline silicon (Si) NPs with diameters ranging from 100 to 200 nm and a narrow size distribution. Crystalline Si exhibits a high refractive index (*n* ∼ 4) and a low extinction coefficient, enabling vivid structural coloration across the visible range. Due to the strong backward scattering resulting from the high refractive index, even a sub‐monolayer of NPs is sufficient to produce bright structural colors [31, 32]. Moreover, a protective overlayer (such as a resin with a refractive index of *n* ∼ 1.5) can be applied without degrading the structural color, owing to the large refractive index contrast. These features are unique to high‐refractive‐index dielectric NPs and are not achievable with low‐refractive‐index materials.

In this work, we develop Si NP‐based inks and demonstrate their applicability to the inkjet structural color printing of complex images. The ink is formulated by dispersing Si NPs coated with thick silica shells (Si@SiO_2_ NPs) into an water‐based acrylic emulsion. We show that vivid structural colors can be produced through inkjet printing and that the hue is widely tunable by adjusting the size of the Si NPs. Furthermore, we demonstrate that the printed images exhibit clearly distinguishable colors in reflection and transmission modes. To elucidate the optimal ink parameters, we first conduct a theoretical analysis of the NP density using Monte Carlo simulations that incorporate single‐particle scattering properties derived from Mie theory. This approach identifies the conditions required to achieve strong reflection/transmission asymmetry across different particle sizes. We then experimentally validate the predicted coloration and optical asymmetry via inkjet printing using the developed Si@SiO_2_ NP/resin inks.

## 2 Simulation of Reflection and Transmission Colors of a Film Containing Si NPs

### 2.1 Forward and Backward Scattering of a Single Si NP

**FIGURE 1:**
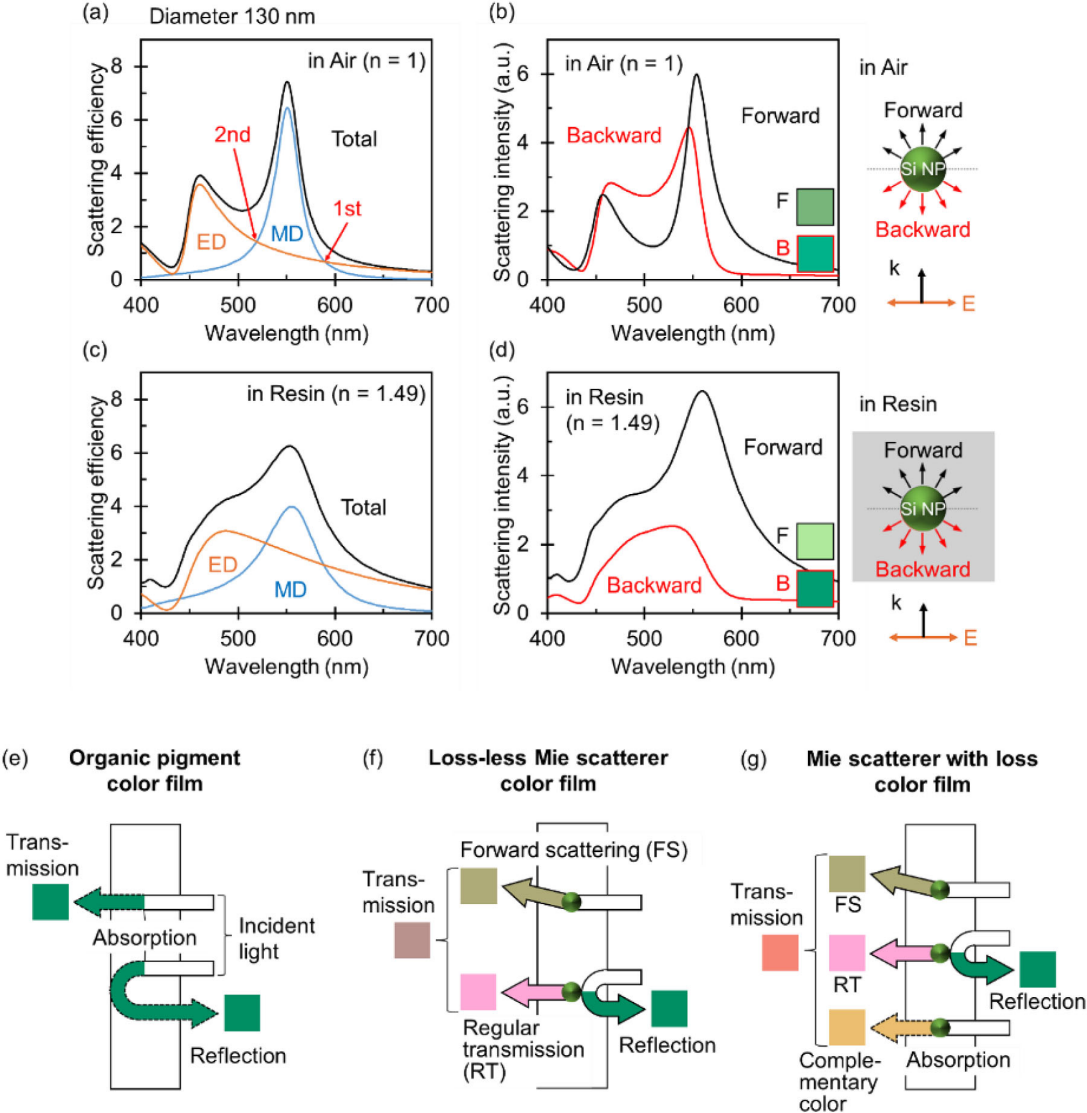
(a,c) Calculated scattering spectra of a single Si NP with a diameter of 130 nm in air (*n* = 1) and in acrylic resin (*n* = 1.49), respectively. Blue, red, and black curves represent the magnetic dipole (MD) mode, electric dipole (ED) mode, and total Mie scattering efficiency, respectively. (b,d) Calculated forward (black curves) and backward (red curves) scattering spectra corresponding to the condition in (a) and (c). The color palettes show the sRGB colors converted from the respective scattering spectra. (e–g) Schematic illustrations of color films containing organic pigments (e), lossless Mie scatterers (f), and Mie scatterers with intrinsic absorption (g).

Figure 1a shows the scattering efficiency spectrum of a single Si NP with a diameter of 130 nm in air. The MD and ED modes are simultaneously excited in a dielectric sphere under plane wave illumination. Due to the high refractive index, the MD and ED modes are spectrally separated. The first and second Kerker conditions, where these modes overlap with equal intensity, are indicated by arrows. At the first Kerker condition, the forward scattering is dominant because the MD and ED modes are in‐phase on the longer wavelength side of the MD mode peak (see Figure S1b). Conversely, a significant phase difference arises in the spectral region between the MD and ED peaks. This results in the destructive interference of forward scattering and the enhancement of backward scattering. Consequently, the backward scattering intensity becomes comparable to, or even exceeds, the forward scattering intensity (the second Kerker condition) [24, 25, 33]. Notably, this distinct scattering asymmetry is observed only in high‐refractive‐index NPs; in low‐index NPs, the MD and ED spectra overlap largely, and their phase difference remains small across the visible range (see Figure S1c,d), leading to dominant forward scattering across all wavelengths [24, 25, 26].

Figure S2 shows the angle‐resolved scattering spectra of a single Si NP with a diameter of 130 nm in air. The spectral shapes differ significantly between the forward and backward hemispheres yet remain highly consistent within each hemisphere. This indicates that the scattering color varies between the forward and backward directions but is uniform within each respective direction. Figure 1b presents the forward and backward scattering spectra derived by integrating the angle‐resolved spectra over the respective hemispheres; the insets show the corresponding perceived colors. A distinct color difference is clearly observed between the forward and backward directions.

In practical applications, inkjet‐printed Si NPs are embedded within a resin matrix. To simulate this configuration, we performed similar calculations for a Si NP embedded in an acrylic resin (polymethyl methacrylate, PMMA; *n* = 1.49). The reduced refractive index contrast broadens the scattering spectrum and lowers the overall scattering efficiency (Figure 1c), resulting in a decrease in backward scattering intensity (Figure 1d). Nevertheless, the backward scattering peak remains distinct, and the resulting color is comparable to that observed in air. This behavior contrasts with that of lower refractive‐index dielectrics such as TiO_2_ (*n* ∼ 2.6), where the backward scattering peak is substantially suppressed due to the much smaller refractive index contrast (Figure S3). Therefore, a sufficiently high refractive index is essential for preserving structural color in resin‐embedded systems.

### 2.2 Origin of Reflection/Transmission Asymmetry

We now address the reflection/transmission asymmetry in films containing NPs (Figure 1e–g). When organic dyes or pigments are dispersed in a transparent film (Figure 1e), light of specific wavelengths is absorbed. This results in the appearance of the same complementary color in both reflection and transmission, as absorption is independent of the light propagation direction. In contrast, when lossless high‐refractive‐index Mie scatterers are dispersed (Figure 1f), the reflection color arises from the backward scattering by the NPs, while the transmission color is determined by the forward scattering combined with the spectral subtraction of the backward scattering. This leads to a reflection/transmission asymmetry. In the case of Si NPs, slight absorption in the blue spectral region also affects the transmission color (Figure 1g). Since backward scattering is relatively unaffected by this absorption, the presence of intrinsic absorption further enhances the reflection/transmission asymmetry.

### 2.3 Monte Carlo Simulation of Reflection and Transmission Colors

**FIGURE 2:**
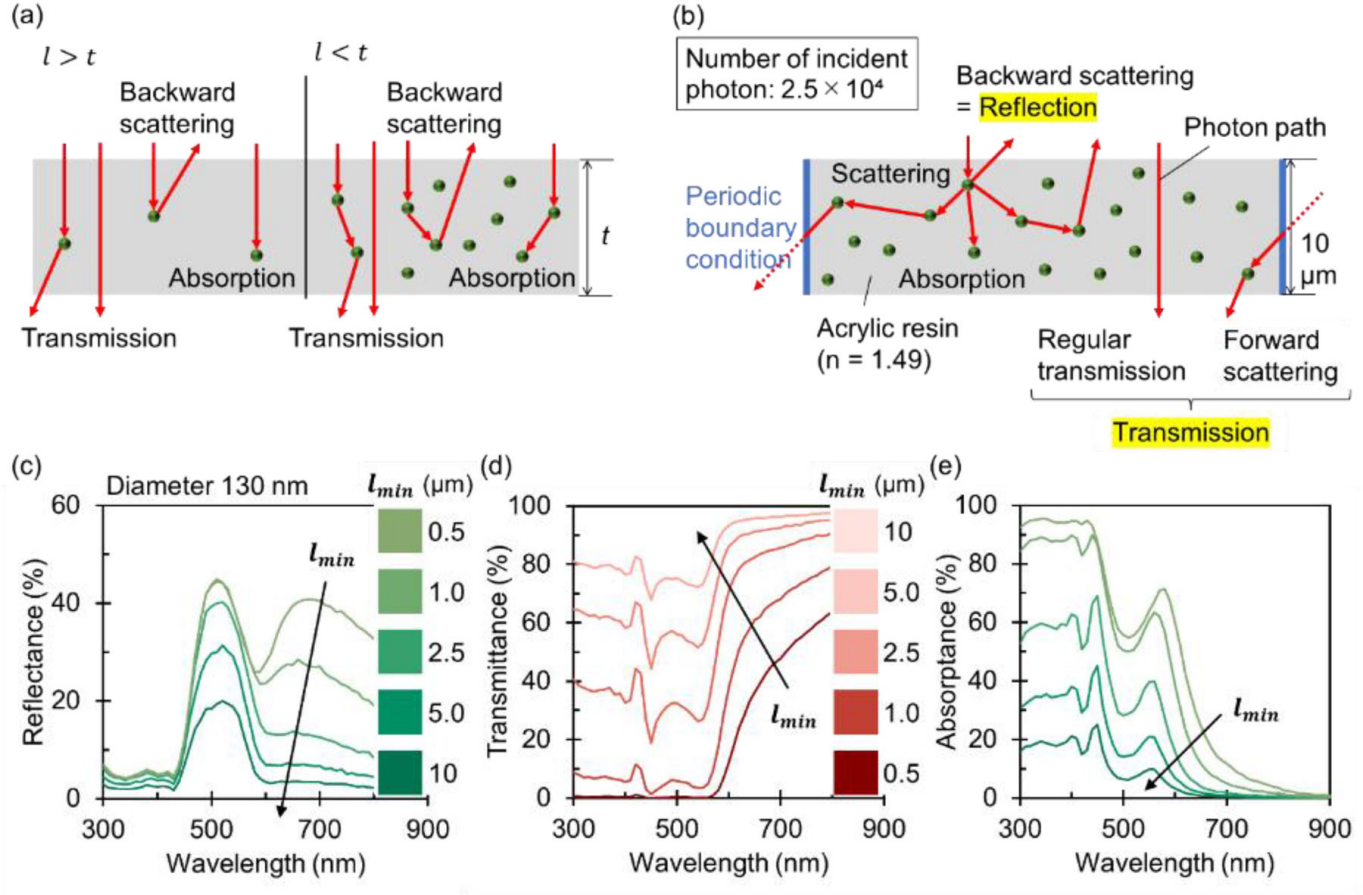
(a) Schematic illustration of photon‐Si NP interactions in two regimes: *l* > *t* (left) and *l* < *t* (right), where *l* is the photon mean free path and *t* is the film thickness. (b) Schematic illustration of the Monte Carlo simulation model. (c–e) Simulated reflectance (c), transmittance (d), and absorptance (e) spectra for minimum mean free paths (*l_min_*) ranging from 0.5 to 10 µm. The Si NP diameter is fixed at 130 nm. The color palettes represent sRGB values converted from the simulated spectra.

In realistic Si NP‐dispersed films, a multitude of Si NPs are randomly distributed. When the photon mean free path (*l*) exceeds the film thickness (*t*)—corresponding to a low Si NP concentration—most incident photons either pass through the film without interaction or undergo only a single scattering event (Figure 2a, left). Under this regime, the reflection color closely resembles that of a single Si NP. Conversely, when *l* < *t*, incident photons undergo multiple scattering events before exiting the film (Figure 2a, right). Since the mean free path is highly wavelength‐dependent (stemming from the Si NP extinction cross‐section), multiple scattering significantly modifies both the reflection and transmission colors.

To quantitatively evaluate these colors, we employ a Monte Carlo simulation [34]. Figure 2b illustrates the simulation model: Si NPs are randomly distributed within a 10 µm‐thick resin film. Periodic boundary conditions were applied in the lateral directions. Photons emerging from the top surface were recorded as reflected, while those emerging from the bottom surface were recorded as transmitted. The transmitted component includes both forward‐scattered photons and ballistic photons (those traversing the film without interaction). The simulation incorporates three stochastic variables: probability of scattering versus absorption upon photon–NP interaction, the scattering angle, and the photon mean free path. Both the interaction probabilities and the scattering angles were determined using Mie theory. The minimum mean free path (*l_min_*) is defined as $l_{min}=1/\sigma_{ext}^{max}N$, where $\sigma_{ext}^{max}$ is the maximum extinction cross‐section within the simulated wavelength range, and *N* is the Si NP number concentration. Near‐field interactions between Si NPs were neglected as *l_min_* remains comparable to or larger than the wavelength.

Figure 2c–e presents the simulated reflectance, transmittance, and absorptance spectra for films containing 130 nm‐diameter Si NPs, with *l_min_* varied from 0.5 to 10 µm. Results for other NP sizes (90–190 nm) are provided in the Figures S4–S6. In Figure 2c, the reflectance spectra exhibit a peak around 500 nm, consistent with the backward scattering spectrum of a single Si NP (Figure 1d). As *l_min_* decreases (i.e., the Si NP concentration increases), this peak intensifies, and a secondary broad peak attributed to multiple scattering emerges at a longer wavelength (>600 nm). To elucidate the origin of this multiple scattering peak, we calculated the absorptance spectra using the relation

$$A\left(\lambda \right)\;\left[\%\right]=100-R\left(\lambda \right)-T\left(\lambda \right)$$

where *R*(*λ*) and *T*(*λ*) denote the reflectance and transmittance spectra, respectively (Figure 2e). The absorptance spectra feature peaks associated with the MD resonance around 560 nm and the magnetic quadrupole (MQ) resonance around 450 nm. The gradual increase in absorption below 400 nm arises from interband transitions in Si NPs. Consequently, the multiple‐scattering peak in reflection originates from the interplay between (i) the long‐wavelength tail of the forward‐scattering spectrum of individual Si NPs (see Figure 1d) and (ii) a reflection dip induced by partial absorption associated with the MD mode. Enhanced multiple scattering increases the overall reflectivity while simultaneously increasing the absorption probability, thereby making the reflectance dip around 600 nm more pronounced (Figure 2c). Consequently, the multiple‐scattering peak exhibits an apparent red‐shift. The growth of the multiple scattering peak shifts the reflection color from pure green to yellow‐green, as shown in the color palettes on the right side of Figure 2c.

Figure 2d shows the corresponding transmittance spectra and the associated color panels. The transmission color is predominantly reddish. Above 600 nm, both absorption and reflection are minimal, contributing to the reddish transmission color. The transmitted light comprises ballistic photons (which bypass interaction with Si NPs) and those forward‐scattered by the NPs. If a forward‐scattered photon is subsequently back‐scattered, it contributes to the multiple scattering peak observed in Figure 2c. As shown in the Figure S7, the forward scattering peak red‐shifts with decreasing *l_min_*, further enhancing the reddish hue in transmission. Additionally, the regular (ballistic) transmittance remains high at wavelengths exceeding 600 nm (Figure S8), reinforcing the reddish transmission color.

**FIGURE 3:**
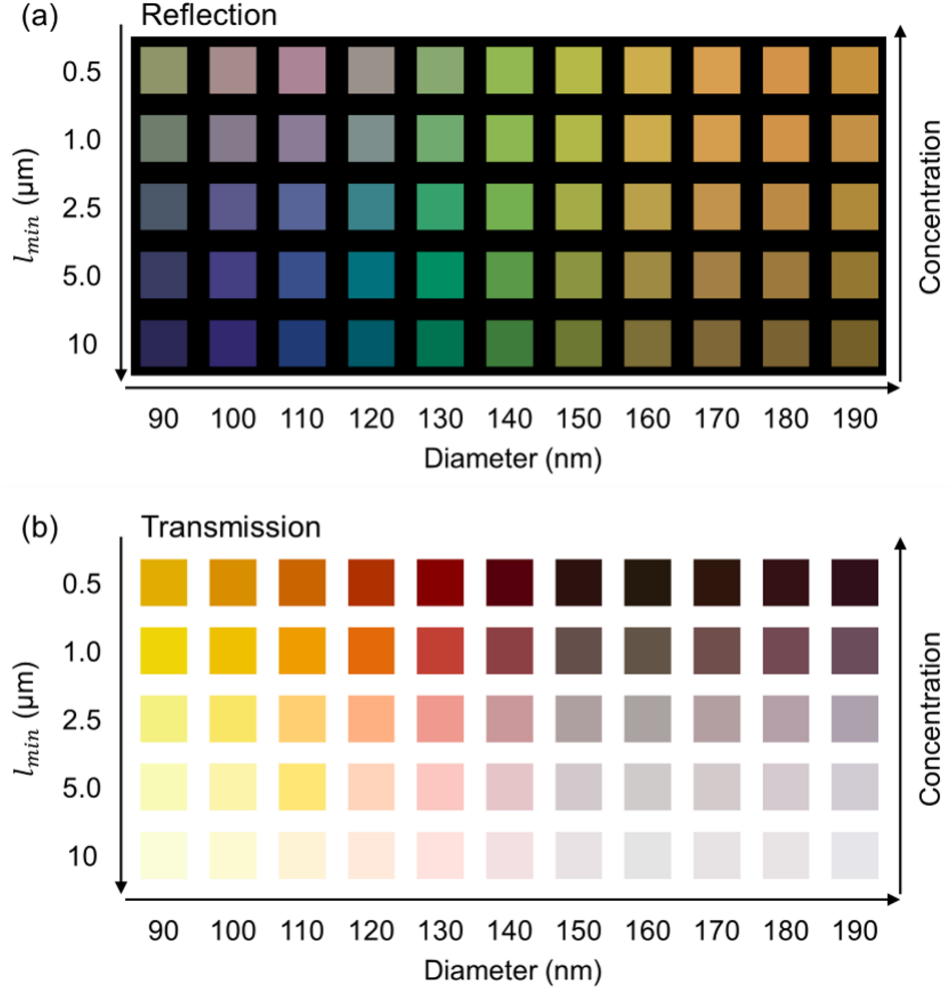
(a,b) sRGB color palettes derived from simulated reflectance (a) and transmittance (b) spectra, calculated for various NP diameters and minimum mean free paths (*l_min_*).

The simulated reflection and transmission color for various values of *l_min_* and Si NP diameters are summarized in Figure 3a,b, respectively. The corresponding spectra are provided in the Figures S4 and S5. For large *l_min_*, the reflection color shifts from violet to orange with increasing particle diameter, reflecting the red‐shift of the Mie resonances. For the small diameters, a decrease in *l_min_* significantly alters the reflection color. In contrast, the reflection color remains nearly unchanged for larger diameters (> 140 nm), as the multiple scattering peak shifts into the near‐IR region and thus has exerts minimal impact on the visible spectrum. The transmission colors shown in Figure 3b differ markedly from their reflection counterparts. Many combinations of reflection/transmission color asymmetry, distinct from a simple complementary spectral relationship, are observed. For example, a green‐red asymmetry is evident for 130 nm‐diameter Si NPs, while a purple‐yellow asymmetry appears for 110 nm‐diameter NPs. These asymmetric color relationships are visually highlighted in the Figure S9a–c.

## 3 Structural Color Printing With Si NPs Using an Inkjet Printer

### 3.1 Fabrication of Si NP Inks

**FIGURE 4:**
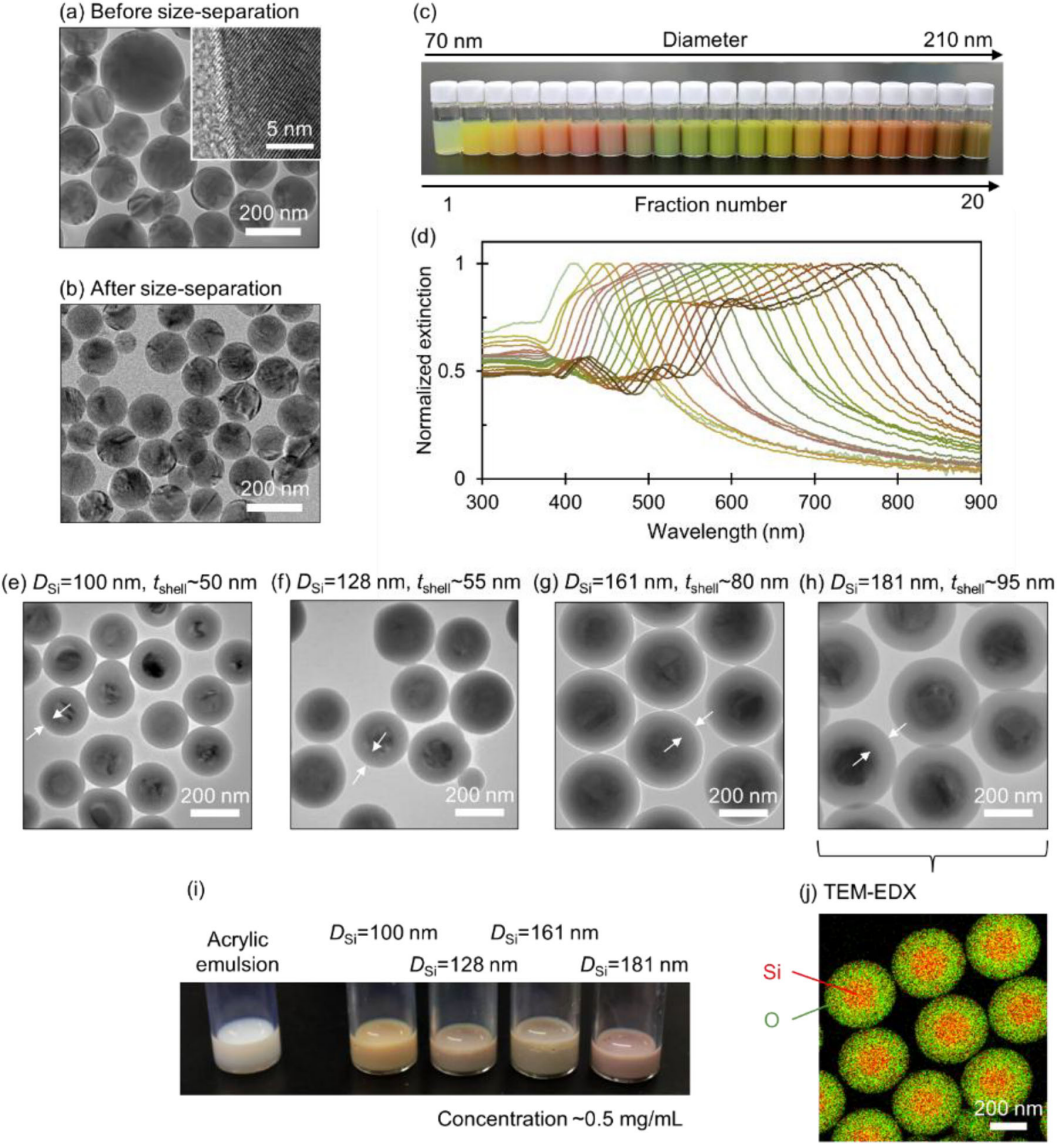
(a) Representative TEM image of as‐synthesized Si NPs. The inset shows a high‐resolution TEM image. Scale bars: 200 nm (main) and 5 nm (inset). (b) TEM image of size‐separated Si NPs with an average diameter of 130 nm. Scale bar: 200 nm. (c) Photographs of colloidal dispersions of size‐separated Si NPs in methanol. (d) Normalized extinction spectra of the size‐separated Si NP colloidal dispersions shown in (c). (e–h) TEM images of Si@SiO_2_ core–shell NPs. The average core diameter (*D*_Si_) are 100 (e), 128 (f), 161 (g), and 181 nm (h), with corresponding shell thicknesses (*t*_shell_) of approximately 50, 55, 80, and 95 nm, respectively. Scale bars: 200 nm. (i) Photograph of the neat acrylic emulsion (left) and the prepared Si NP inks (right) corresponding to (e‐h). The Si NP concentration is 0.5 mg/mL. (j) TEM‐EDX elemental map of a Si@SiO_2_ NP with *D*_Si_ = 181 nm. Red and green indicate silicon (Si) and oxygen (O), respectively. Scale bar: 200 nm.

Since the diameter of Si NPs is much smaller than the nozzle diameter of conventional inkjet systems (typically several tens of µm), these particles are highly suitable for inkjet printing [35]. We therefore developed water‐based Si NP inks compatible with standard inkjet printers. Spherical crystalline Si NPs synthesized by a previously reported method were used as the starting material [31]. Figure 4a presents a transmission electron microscope (TEM) image of the as‐synthesized Si NPs. The particles exhibit a highly spherical morphology. The lattice fringes observed in the high‐resolution inset image correspond to the {111} planes of crystalline Si, confirming the high crystallinity of the NPs. To realize vivid structural colors from Si NPs, the size distribution of NPs should be significantly narrowed. We employed a density gradient centrifugation process to size‐separate the Si NPs (see Experimental Section for details) [31, 36]. Figure 4b shows a TEM image of the Si NPs after size separation. We find a significant narrowing of the size distribution compared to that in Figure 4a.

Figure 4c displays the photographs of colloidal dispersions of size‐separated Si NPs with average diameters (*D*_ave_) ranging from 70 to 210 nm, along with their corresponding extinction spectra (Figure 4d). As *D*_ave_ increases, the Mie resonance peaks red‐shift, resulting in a noticeable color change. The values of *D*_ave_, size distribution, and concentration were determined by fitting the extinction spectra using Mie theory, assuming a Gaussian size distribution (for the detailed procedure and retrieved values, see Figures S10 and S11) [37, 38]. The coefficient of variation (CV)—defined as the standard deviation of the size distribution divided by *D*_ave_—remains below 10% across the entire size range and drops to approximately 5% around *D*_ave_ = 200 nm. The narrow size distribution minimizes color degradation arising from size variations (see Figure S12).

During the solvent evaporation process following inkjet printing, Si NPs tend to aggregate. Direct interparticle contact modifies the Mie resonances and significantly degrades the coloration (Figure S13). To prevent such direct contact, a silica (SiO_2_) shell was formed on the surfaces of the Si NPs (see the Experimental Section for the process). Figure 4e–h shows TEM images of the Si NPs coated with SiO_2_ shells (Si@SiO_2_ NPs), and Figure 4j presents a TEM‐energy dispersive X‐ray spectroscopy (TEM‐EDX) map of Si@SiO_2_ NPs with a core diameter of 181 nm, confirming the formation of a uniform shell. Since the refractive index of SiO_2_ (*n* ∼ 1.46) is comparable to that of acrylic resin (*n *= 1.49), the shell exerts a negligible impact on the scattering properties of the Si NPs (Figure S14). Consequently, the core‐size‐dependent variation in shell thickness observed in Figure 4e–h does not significantly affect the scattering properties. Furthermore, the increased particle size due to the shell does not restrict the density of Si cores in the printed resin matrix, given the small volume fraction of Si@SiO_2_ NPs (< 8 vol%) in the matrix.

We employed a commercial aqueous silicon‐acrylic emulsion (∼40 wt.%; Asahipen Water‐Based Multi‐Purpose Color, Transparent) as the binder. Since this resin itself is water‐insoluble, the liquid emulsion appears milky white (Figure 4i) due to light scattering; however, it forms a transparent film upon drying. The commercial emulsion was diluted with Milli‐Q water to yield an optimal viscosity that balances printed dot morphology with the jetting stability of the printhead. The optimization procedure is detailed in the Section S7. The optimal formulation was obtained when the commercial emulsion was diluted to a solid content of 16 wt.%. Subsequently, the Si@SiO_2_ NPs were dispersed into the diluted emulsion at concentrations of 0.5, 1.0, and 2.0 mg/mL, corresponding to *l_min_* values of 2.5–5.0 µm in the printed film. Note that the concentration values represent the mass of the Si cores (excluding the SiO_2_ shells) per milliliter of dispersion. Figure 4i shows a photograph of the prepared Si NP inks. Due to the turbidity of the resin emulsion, the inks appear less vibrant than the corresponding alcoholic dispersions shown in Figure 4c. Nevertheless, each ink exhibits a distinct color depending on the Si NP size. The observed colors originate primarily from multiple scattering, as the ink bottle diameter (several millimeters) places the system in the *l* << *t* regime depicted in Figure 2a (where *t* represents the bottle diameter). After inkjet printing, however, the film thickness is reduced to a few micrometers, shifting the system to the *l* > *t* regime (Figure 2a). Consequently, the printed films are expected to exhibit the Mie scattering colors characteristic of single Si NPs.

### 3.2 Inkjet‐Printing of Si NPs

**FIGURE 5:**
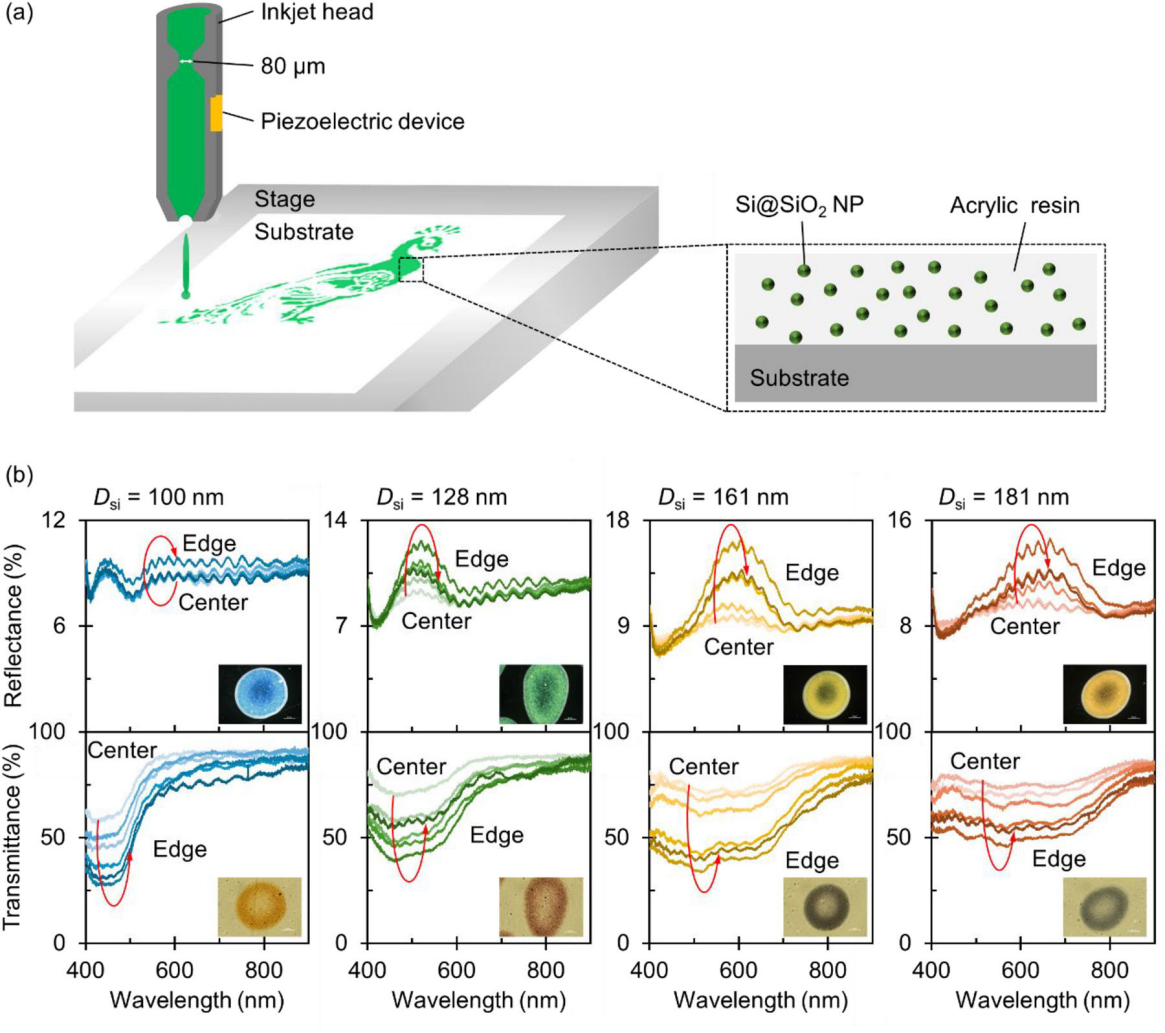
(a) Schematic illustration of the inkjet printing process using a piezo‐driven single‐head inkjet printer. (b) Measured reflectance (top) and transmittance (bottom) spectra of an inkjet‐printed dot composed of Si@SiO_2_ NPs and acrylic resin. The spectral fringes originate from interference at the resin‐air and resin‐substrate interfaces. The core Si NP diameters (*D*_Si_) are 100, 128, 161, and 181 nm, respectively. The Si NP concentration is 2.0 mg/mL (calculated based on the Si core mass). Pale and dark lines indicate spectra measured at the center and the periphery of the dot, respectively. The inset displays optical microscope images of the printed dots.

We employed a drop‐on‐demand piezo‐driven inkjet printer (Microjet LaboJet‐600) equipped with a single print head designed for high viscosity and high surface tension (Microjet IJHD‐1000) to print Si NPs (Figure 5a). Polyethylene terephthalate (PET; Toray Lumirror #125‐T60) was used as the substrate. The optimization procedure for inkjet parameters is detailed in the Section S8. The insets of Figure 5b display microscope images of single printed dots containing Si@SiO_2_ NPs of different sizes, corresponding to those shown in Figure 4e–h. The printed dots exhibit vivid reflection colors that depend on the particle size. Transmission colors, which are distinct from the reflection colors, are also observed. Due to the coffee‐ring effect, the film thickness is non‐uniform within each dot, resulting in a visible contrast between the central and peripheral regions. This spatial variation is evident in the reflectance and transmittance spectra measured at different positions within each dot (Figure 5b; details of the measurement setup are provided in the Experimental Section and Section S9). From the center toward the edge, the reflectance first increases and then decreases, while the transmittance exhibits the opposite trend. Notably, despite the spatial variations of reflectance and transmittance intensity within a dot, the spectral shapes remain remarkably consistent. This indicates that single‐particle Mie scattering properties are preserved even in the particle‐dense peripheral regions, confirming that the SiO_2_ shells effectively prevent near‐field coupling between Si NPs. Indeed, scattering images of individual Si NPs are clearly visible even in the peripheral region under dark‐field optical microscopy (see Section S10).

**FIGURE 6:**
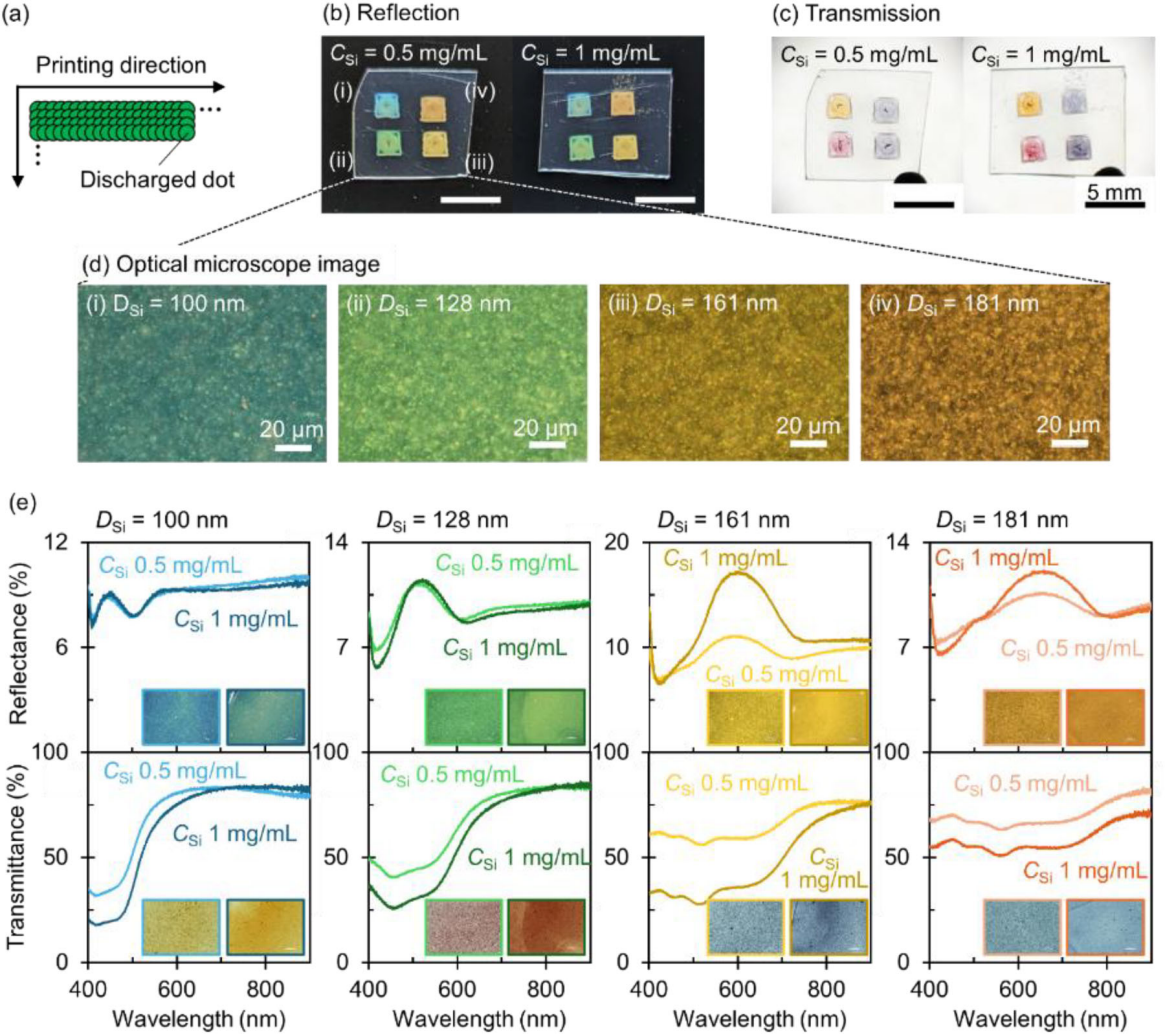
(a) Schematic illustration of the inkjet printing process used to fabricate square patterns with Si NP inks. (b,c) Photographs of the printed substrates in reflection (b) and transmission (c). The Si NP concentrations (*C*_Si_) are 0.5 mg/mL (left) and 1.0 mg/mL (right). The average Si NP diameters (*D*_Si_) are 100 (i), 128 (ii), 161 (iii), and 181 nm (iv). Scale bar: 5 mm. (d) Dark‐field optical microscope images of the printed films shown in (b). Scale bar: 20 µm. (e) Measured reflectance (top) and transmittance (bottom) spectra of the printed films with varying NP diameters and concentrations. The inset displays optical microscope images of the films with *C*_Si_ = 0.5 mg/mL (left) and 1.0 mg/mL (right).

We fabricated 2 × 2 mm square patterns by overlapping adjacent inkjet droplets as illustrated in Figure 6a. Figure 6b,c displays reflection and transmission images of the printed squares (for the photography setup, see Section S9). Both reflection and transmission colors are clearly visible under ambient lighting conditions. In the microscope images shown in Figure 6d, scattering from individual NPs is clearly visible. Figure 6e presents the reflection and transmission spectra of the printed squares. The reflectance of the PET substrate (∼11%) contributes to a background signal to the measured spectra. In the reflectance spectra, peaks originating from Mie scattering at the second Kerker condition are observed, with minimal influence from multiple scattering. For Si NPs with diameters of 100 and 128 nm, the reflectance is nearly independent of the ink concentration. This behavior arises because the Mie resonances for these particles overlap with the intrinsic absorption band of Si, and consequently, the absorption limits the increase in the reflectance at higher concentrations. In contrast, for larger Si NPs (161 and 181 nm), the reflectance peaks lie in spectral regions with lower absorption, allowing the reflectance to increase with raising the concentration. Unlike the reflectance, the transmittance spectra exhibit a distinct concentration dependence across all particle sizes. Transmittance decreases as concentration increases, attributed to enhanced absorption. This trend is consistent with the simulation results (Figures S4–S6).

**FIGURE 7:**
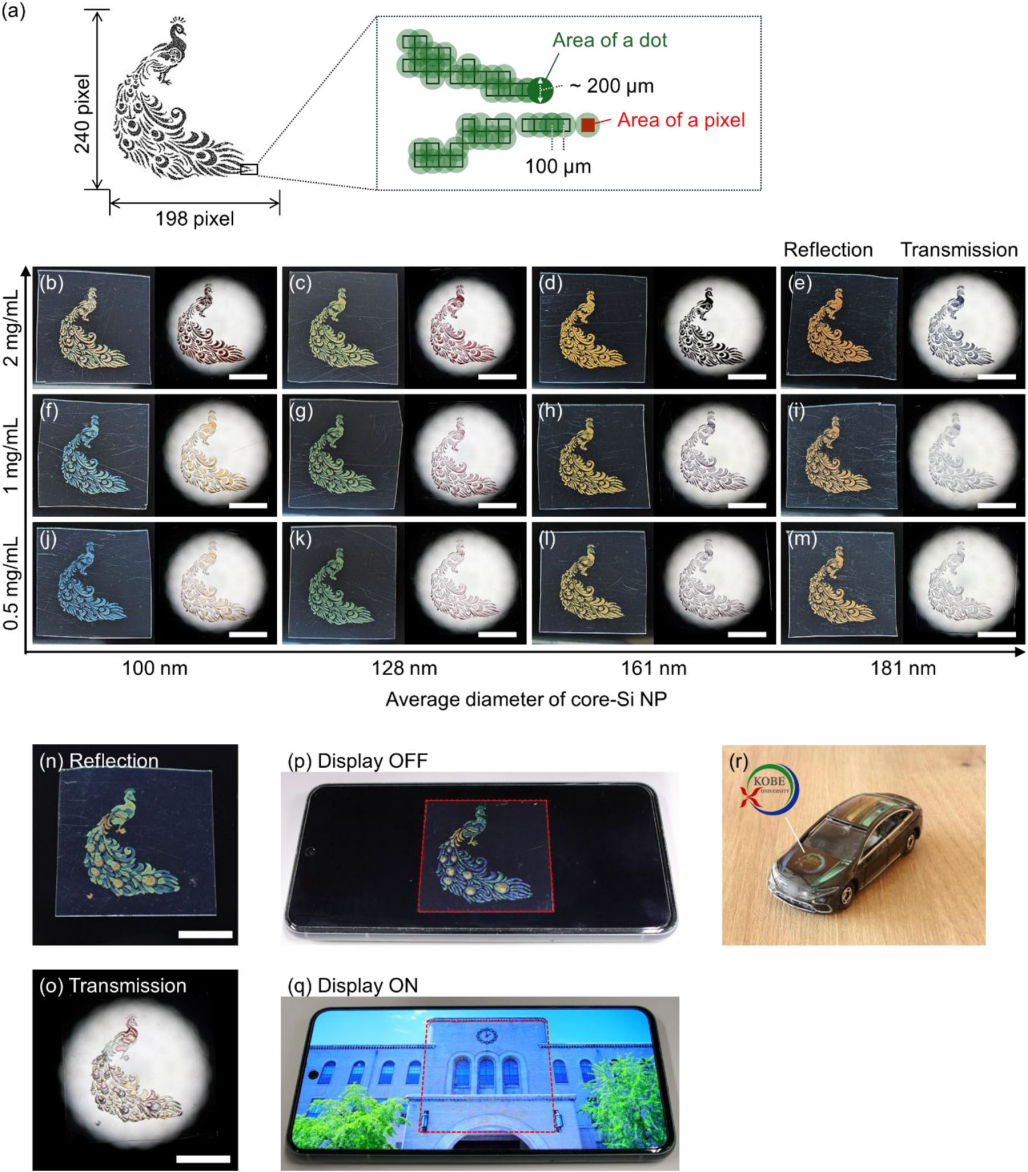
(a) Monochromatic bitmap source image of a digitally designed peacock pattern (derived from a royalty‐free image). The enlarged view illustrates the mapping between image pixels and printed dots. (b‐m) Reflection (left panels) and transmission (right panels) images of peacock patterns printed using Si NP inks with varying diameters and concentrations. The average Si core diameters (*D*_Si_) are 100 nm (b,f,j), 128 nm (c,g,k), 161 nm (d,h,l), and 181 nm (e,i,m). The Si NP concentrations (*C*_Si_) are 2.0 mg/mL (b–e), 1.0 mg/mL (f–i), and 0.5 mg/mL (j–m). Scale bar:1 cm. (n,o) Multicolor reflection (n) and transmission (o) images of the peacock pattern, printed using the same ink formulations as in (j–m). The dot‐to‐dot pitch is 100 µm. Scale bar:1 cm. (p,q) Photographs of the multicolor printed film placed over a smartphone display in the off‐state (p) and on‐state (q). The ink composition corresponds to that in (j–m), but with a dot‐to‐dot pitch of 200 µm. (r) Photograph of a toy car printed with the university logo (reproduced with permission from Kobe University). The car dimensions are approximately 7.5 cm (length) × 2.5 cm (height). The ink composition is identical to that used in (j–m).

To demonstrate the practical applicability of the Si NP inks, we printed a digitally designed pattern using inkjet printing. A monochromatic bitmap image consisting of 198 × 240 pixels was used as the source (Figure 7a). The printer ejected droplets at pixels corresponding to a bit value of 1 (black regions in Figure 7a). A dot‐to‐dot pitch of 100 µm was selected to ensure overlap between the printed dots, which had a diameter of approximately 200 µm, as depicted in the schematic illustration. Figure 7b–m displays reflection and transmission photographs of the patterns printed using Si NP inks with various diameters and concentrations. The fine features of the design were faithfully reproduced, with the resulting colors varying according to both the particle size and the ink concentration. Notably, a distinct color asymmetry was observed between reflection and transmission modes. At low Si NP concentrations, vivid reflection colors were achieved due to minimal multiple scattering, whereas transmission colors appeared pale (Figure 7j–m). In contrast, at higher concentrations, the transmission colors became more pronounced, while the saturation of the reflection colors decreased due to enhanced multiple scattering (Figure 7b–e). To fabricate a multicolor image, we sequentially printed different patterns onto a single substrate using Si NP inks with diameters of 100 nm (blue), 128 nm (green), 161 nm (yellow), and 181 nm (orange) (for detailed procedure, see Section S11). Figure 7n,o presents the resulting multicolor reflection and transmission images, respectively. These results demonstrate that distinct and asymmetric multicolor images can be produced using Si NPs of various diameters.

The combination of vivid reflection and faint transmission at low concentrations offers significant potential for overt/covert security features and decorative overlays on digital displays. These properties can be leveraged for anti‐counterfeiting measures [5, 39, 40, 41, 42]. To demonstrate this concept, we printed a multicolor pattern designed to exhibit faint transmission colors. The dot‐to‐dot pitch was increased to 200 µm to reduce the dot overlap. The Si NP concentration was adjusted to 0.5 mg/mL, a condition confirmed to yield faint transmission colors (Figure 7j–m). As demonstrated in Figure 7p,q, when the printed film is placed over a digital display, the printed image is clearly visible in the display's off‐state due to vivid reflection (Figure 7p). However, it becomes virtually invisible in the on‐state due to the high transparency of the film (Figure 7q). Such films enable the display of static images or advertisements when the screen is powered off, providing a zero‐energy information display. Finally, Figure 7r presents an example of 3D printing capability: the Kobe University logo and colored stripes were printed onto a black toy car, demonstrating the applicability of Si NP inks for decorating three‐dimensional objects.

Finally, we summarize the advantages of Si NP‐based structural color pigments compared to conventional inkjet pigments. The Si NP‐based pigments are intrinsically non‐fading and stable in harsh environments, eliminating concerns regarding color degradation. Furthermore, the inherent reflection/transmission asymmetry arising from Mie resonances enables the design of multicolor dichroic prints, offering functionalities unavailable with conventional pigments. Currently, however, the color gamut of Si NP inks is not as broad as that of conventional inkjet pigments. Yet, this is not a fundamental limitation of dielectric nanostructures; wider color gamut have been demonstrated in nanostructures fabricated via top‐down methods such as electron beam lithography [13, 19]. Therefore, expanding the color gamut of Si NP inks to match that of lithographically produced nanostructures remains a key objective for future research.

## 4 Conclusion

In this work, we demonstrated the inkjet printing of Si NP inks and elucidated the mechanism behind their inherent color asymmetry in reflection and transmission. Analytical calculations confirmed that individual Si NPs exhibit directional scattering asymmetry due to the Kerker effect. Furthermore, owing to their high refractive index (*n* ∼ 4), Si NPs retain vivid Mie‐resonant colors even when embedded in a resin matrix, which is unattainable with moderate‐index (*n *< 3) materials such as TiO_2_. We investigated the reflection and transmission properties of Si NP‐dispersed films using Monte Carlo simulations. These simulations revealed that the color asymmetry between reflection and transmission arises from the interplay between the Kerker effect and optical absorption enhanced by multiple scattering. To experimentally validate these findings, we developed water‐based Si NP inks and fabricated structural color films via inkjet printing. The resulting films exhibited vivid Mie scattering colors and pronounced reflection/transmission asymmetry. We successfully produced multicolor patterns with tailored asymmetry by employing Si NPs of various diameters. This work paves the way for scalable structural coloration technologies based on Si NPs, enabling multicolor dichroic decorations for applications in art, anti‐counterfeiting, and semi‐transparent smart windows.

## 5 Experimental Methods

### 5.1 Analytical Calculations

Scattering spectra of a single Si NP and a TiO_2_ NP were calculated analytically using MATLAB based on Mie theory [22, 43]. Forward scattering intensities were obtained by integrating the scattering intensity over the angular range of 0°–90° relative to the incident direction, while backward scattering was integrated from 90° to 180°. The refractive indices of crystalline Si and TiO_2_ (anatase) were obtained from the literature [44, 45].

### 5.2 Monte Carlo Simulations

The simulation model consists of a resin film containing randomly distributed Si NPs. The unit cell dimensions were set to 50 µm in both the *x*‐ and *y*‐directions, and 10 µm in the *z*‐direction. Incident light propagated along the *z*‐axis. Periodic boundary conditions were applied along the *x*‐ and *y*‐axes to simulate a laterally infinite film with a thickness of 10 µm. The refractive index of the host material (PMMA) was assumed to 1.49, while that of Si was obtained from the literature [44]. Simulations were performed over a wavelength range of 300–900 nm at 10 nm intervals, using 25 000 incident photons. Further details regarding the simulation method can be found in our previous publication [34].

### 5.3 Fabrication of Si NPs

Silicon monoxide (SiO) flakes (10 g, several millimeters in size) were placed in an alumina crucible and annealed at 1475°C–1500°C for 30 min under a nitrogen atmosphere using an electric furnace (HF‐1700N, SK Medical Electronics). The annealed samples consisted of Si NPs embedded within a SiO_2_ matrix. The SiO_2_ matrix was removed via chemical etching; 1.5 g of the annealed sample was dispersed in 25 mL of hydrofluoric acid (HF, concentration 48 wt.%) and treated in an ultrasonic bath for 60 min. The resulting colloidal dispersion was washed several times with methanol to remove residual HF. Further details regarding the fabrication conditions of Si NPs are available in our previous publication [31].

### 5.4 Size Separation of Si NPs

Si NPs were size‐separated using density gradient centrifugation, as previously reported by our group [31, 36]. A centrifugal tube was filled to half its volume with a 50 wt.% aqueous sucrose solution, followed by the careful addition of a 20 wt.% sucrose solution. A continuous density gradient (ranging from 50 wt.% at the bottom to 20 wt.% at the top) was generated using Gradient Master 108 (BioComp). A dispersion of Si NPs in methanol (∼ 40 mg/mL, 2.1 mL) was layered onto the gradient, and the tube was centrifuged in a swing‐bucket rotor at 2500 × *g* for 70 min. After centrifugation, the solution was fractionated in 3 mm increments from the top using the Piston Gradient Fractionator (BioComp). The collected fractions were washed with Milli‐Q water to remove sucrose and subsequently redispersed in methanol, yielding size‐separated colloidal Si NP suspensions.

### 5.5 Formation of SiO_2_ Shells

SiO_2_ shells were fabricated using the Stöber method. Milli‐Q water (1.8 mL) and ammonia solution (28 wt.%, 2.7 mL) were added to a Si NP colloidal dispersion, prepared by dispersing 8 mg of Si NPs in ethanol (total volume adjusted to yield a concentration of 0.6 mg/mL). The mixture was stirred at 800 rpm at room temperature for 5 min. Subsequently, 1.1 mL of tetraethyl orthosilicate (TEOS, diluted in ethanol to 25 vol.%) was added to the reaction mixture, and stirring continued for an additional 1 h. The resulting particles were washed with ethanol to remove byproduct SiO_2_ NPs and residual NH_3_, yielding core–shell Si@SiO_2_ NPs. The shell thicknesses were confirmed via transmission electron microscopy (TEM; JEM‐120i, JEOL). The measured shell thicknesses were 45–55 nm for 100 nm Si NPs, 50–60 nm for 128 nm NPs, 75–85 nm for 161 nm NPs, and 90–100 nm for 181 nm NPs. Elemental mapping was performed using scanning transmission electron microscopy (STEM; JEM‐2100F, JEOL) equipped with an energy‐dispersive X‐ray spectrometer (EDX; JED‐2300, JEOL).

### 5.6 Optical Characterization of Printed Films

Reflectance and transmittance spectra were measured via microspectroscopy using an inverted optical microscope (Ti‐U, Nikon). For reflectance measurements, the sample was placed face‐down on the stage and illuminated from below using a halogen lamp through a 50× objective lens (NA = 0.8) in bright‐field mode. The reflected light was collected by the same objective, directed to the entrance slit of a monochromator (Kymera 328i, Andor; slit width: 300 µm), and detected by an electronically cooled CCD (Newton, Andor). Reflection images were captured using the same optical configuration in dark‐field mode using a color CMOS camera (DS‐Fi3, Nikon). For transmittance measurements, the sample remains in the face‐down position and is illuminated from above using a halogen lamp. The transmitted light was collected by the 50× objective lens (NA = 0.8) positioned beneath the sample, then directed to the monochromator and CCD following the same detection path as the reflectance measurements. Transmission images were acquired using the color CMOS camera under the same illumination conditions. Schematic illustrations of the measurement setups are provided in Figure S17.

### 5.7 Statistical Analysis

The color palettes presented in Figures 2, 3, and Figure S9 were obtained via the following procedure. First, the reflectance and transmittance spectra shown in Figures S4 and S5, respectively, were converted to XYZ tristimulus values and subsequently to sRGB values. The assumed light source was CIE standard illuminant D65. Subsequently, the sRGB values were gamma‐corrected with a value of *γ* = 2.4. The final color palettes were obtained by converting these modified values into their corresponding colors. The CIE chromaticity plots shown in Figure S9 were derived by converting the XYZ values into chromaticity coordinates (*x*, *y*).

The color palettes in Figure 1 and Figures S2, and S3 were obtained by first normalizing the corresponding scattering spectra and then converting them to XYZ values; the subsequent processing steps were identical to those described above. Similarly, the CIE chromaticity plots in Figure S12 were obtained by converting the XYZ values to chromaticity coordinates.

The extinction spectra shown in Figure 4d were obtained by normalizing the measured data. The average Si NP diameter, size distribution, and concentration were determined by fitting the calculated spectra with measured extinction spectra. The mean squared error (MSE) of the fitting was below 0.004 within the diameter range of 100–200 nm. Details of the fitting procedure are provided in Section S5.

The reflectance spectrum (*R*) shown in Figures 5,6, and Figure S13 was obtained using the following equation:

$$R=\frac{I_{sample}-I_{dark}}{I_{background}-I_{dark}}\times R_{substrate}$$

where *I_sample_* and *I_background_* are measured reflected‐light intensities of a sample and a substrate, respectively, and *I_dark_* is a dark current (measured in the absence of illumination). *R_substrate_* denotes reflectance of the substrate, which was 11% based on the product datasheet (Lumirror #125‐T60, Toray). The transmittance spectra were obtained via an analogous process using the substrate transmittance of 89%.

## Conflicts of Interest

The authors declare no conflicts of interest.

## Supporting information




**Supporting File**: adma72691‐sup‐0001‐SuppMat.docx.

## Data Availability

The data that support the findings of this study are available from the corresponding author upon reasonable request.
