# Supplementary material for: Structural Color Inkjet Printing With Mie‐Resonant Silicon Nanoparticles
Source: Adv Mater. 2026 Apr 3;38(25):e23036. doi: 10.1002/adma.202523036 (PMC13137761; doi:10.1002/adma.202523036)
Supplement: Supplementary file 1 — Supporting File: adma72691‐sup‐0001‐SuppMat.docx. [file ADMA-38-e23036-s001.docx]

Supporting Information

**Structural Color Inkjet Printing with Mie-Resonant Silicon Nanoparticles**

Hiroto Yamana, Haruki Tanaka, Hiroshi Sugimoto*, and Minoru Fujii*

**1. Scattering and phase spectra of a Si NP and a low-refractive-index NP (*n*_NP_ = 2.0)**


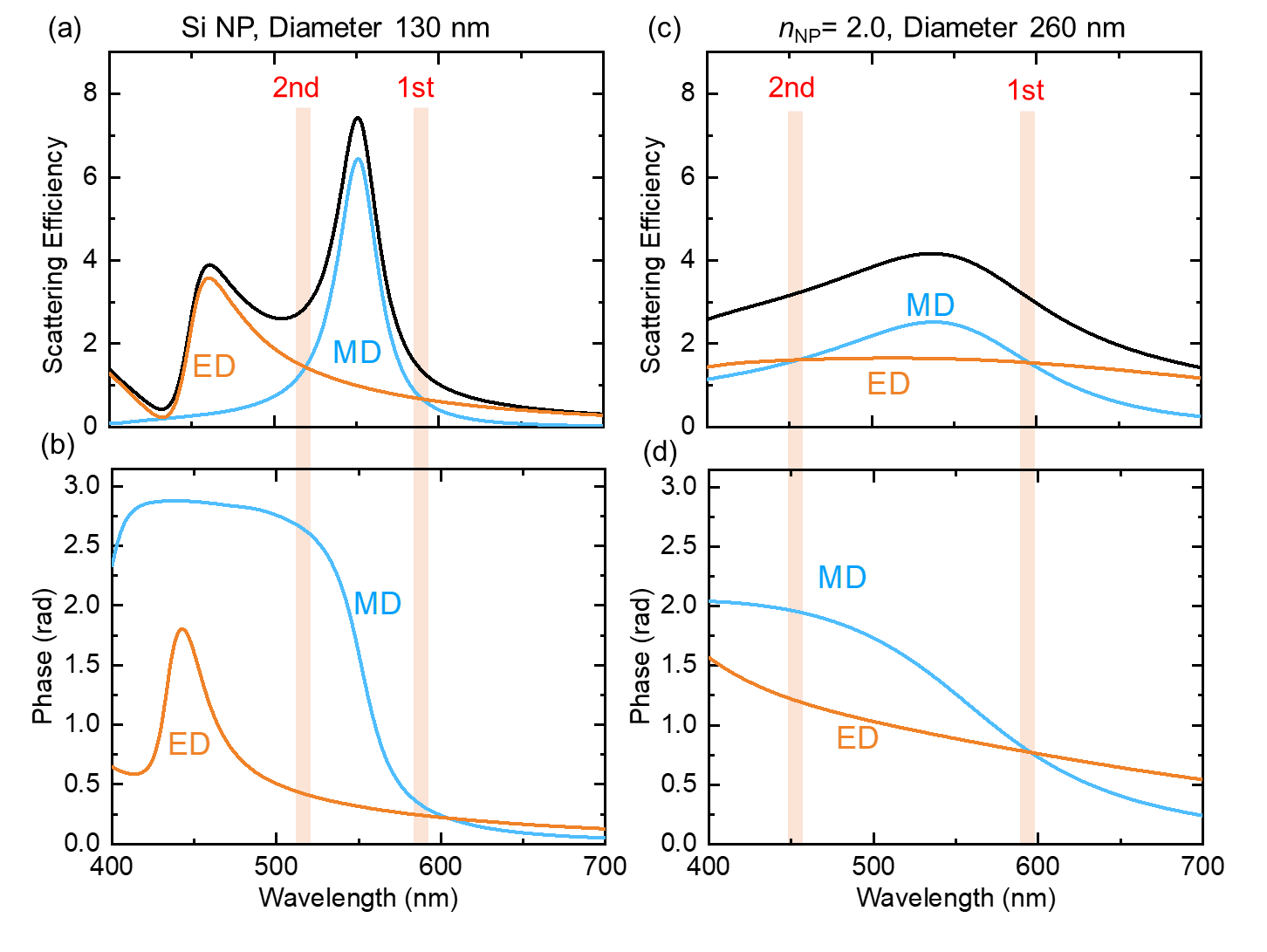


**Figure S1.** (a, c) Calculated scattering spectra (black curves) of a Si NP (a) and a low-index NP (*n*_NP_ = 2.0) (c) with diameters of 130 nm and 260 nm, respectively. The blue and orange curves represent the contribution of the magnetic dipole (MD) and electric dipole (ED) modes, respectively. (b, d) Calculated phase spectra of the MD (blue curves) and ED (orange curves) modes for a Si NP (b) and a low-index NP (*n*_NP_ = 2.0) (d) with diameters of 130 nm and 260 nm, respectively. All calculations were performed in air (*n* = 1).

**2 Angle-resolved scattering spectra of a Si NP**


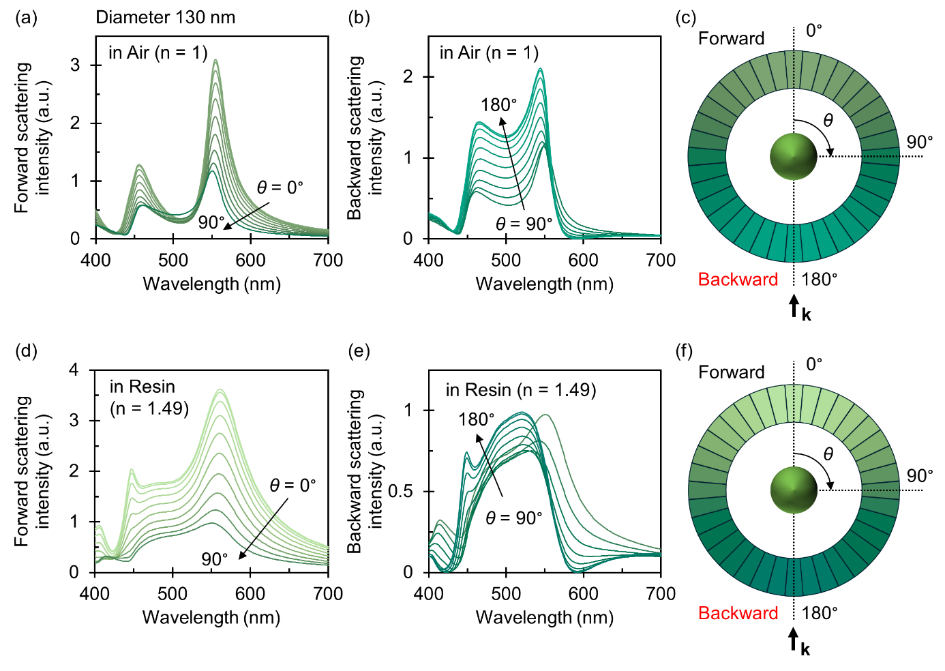


**Figure S2.** (a, b) Calculated scattering spectra of a Si NP with a diameter of 130 nm in air (*n* = 1) for the forward (a) and backward (b) directions. The scattering angle (*θ*) relative to the incident direction was varied from 0° to 90° for the forward scattering and from 90° to 180° for the backward scattering in 10° increments. (c) Corresponding perceived colors derived from the spectra in (a) and (b). (d, e) Calculated scattering spectra of a Si NP with a diameter of 130 nm in resin (*n* = 1.49) for the forward (d) and backward (e) directions. (f) Corresponding perceived colors derived from the spectra in (d) and (e). The scattering spectra and corresponding perceived colors differ between the forward (*θ* = 0°-90°) and backward (*θ* = 90°-180°) directions　while they are similar within each range.

**3. Scattering spectra of a TiO₂ NP in air and in resin**


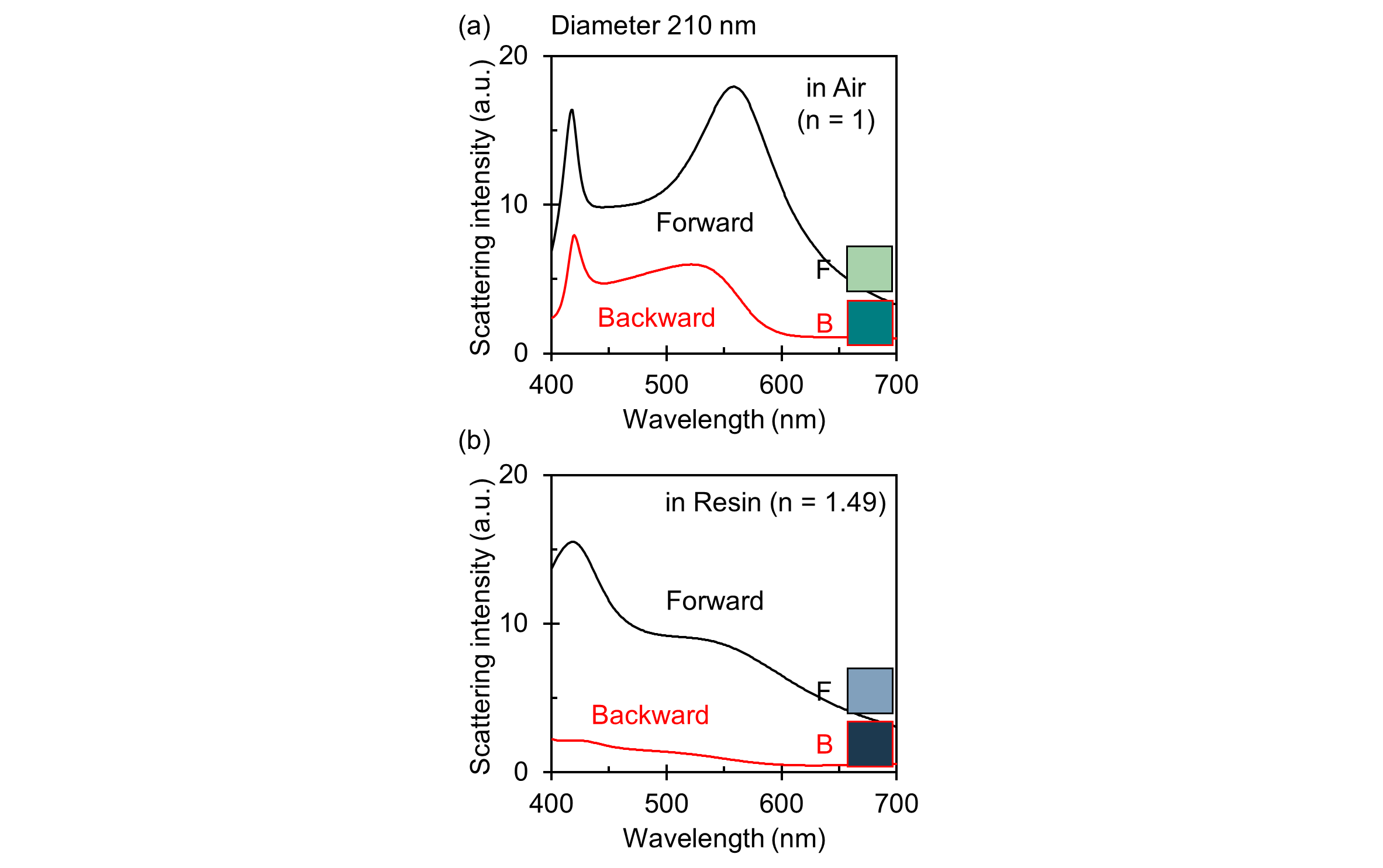


Figure S3. Calculated forward (black curve) and backward (red curve) scattering spectra of a single TiO₂ NP with a diameter of 210 nm in air (*n* = 1) (a) and in resin (*n* = 1.49) (b). The color palettes represent the corresponding sRGB colors derived from the scattering spectra. Due to the relatively low refractive index of TiO₂ (*n* ~ 2.6), the NP fails to maintain distinctive backward scattering peaks in resin, resulting in a significant color change from that in air.

**4. Simulated optical properties of Si NP-dispersed resin films**


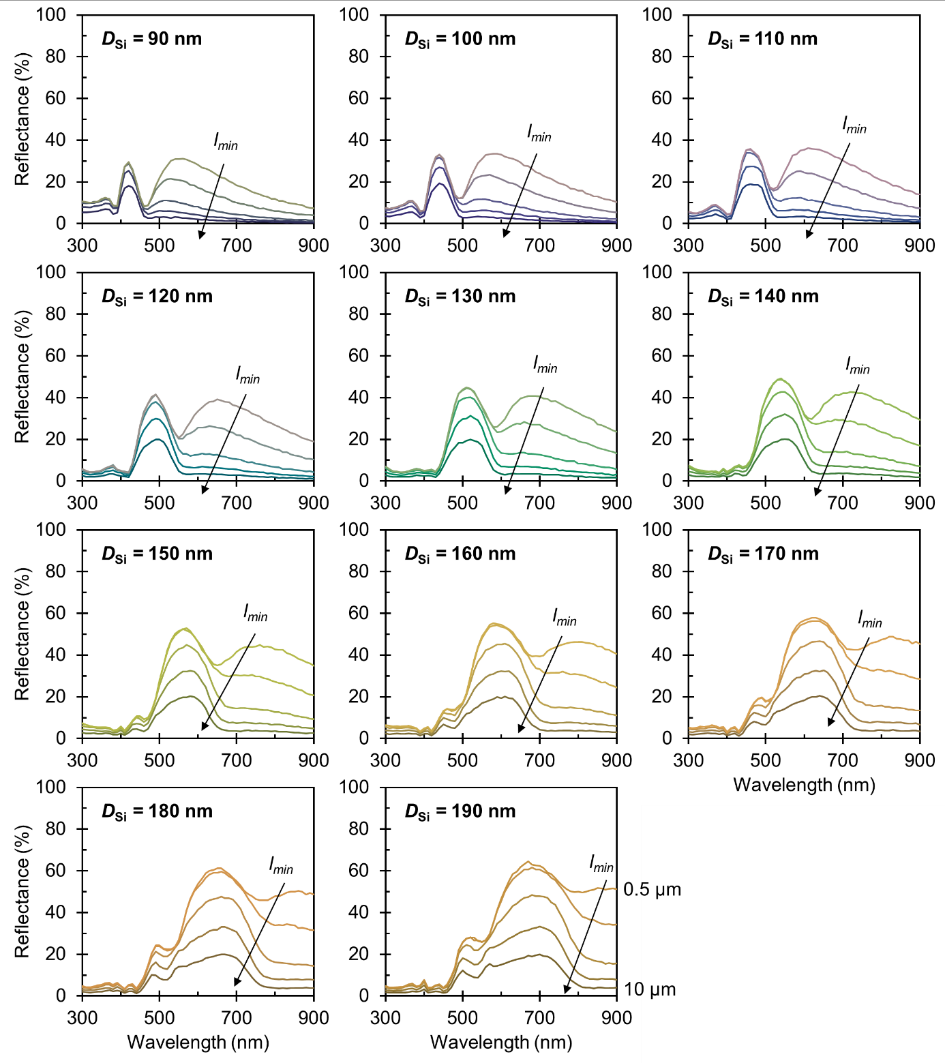


Figure S4. Simulated reflectance spectra of Si NP-dispersed resin films with Si NP diameters ranging from 90 nm to 190 nm. The minimum mean free paths (*l_min_*) of photons were set to 0.5, 1.0, 2.5, 5.0, and 10 µm. The color palettes representing reflection colors shown in Figure 3a of the main text were derived from these spectra.


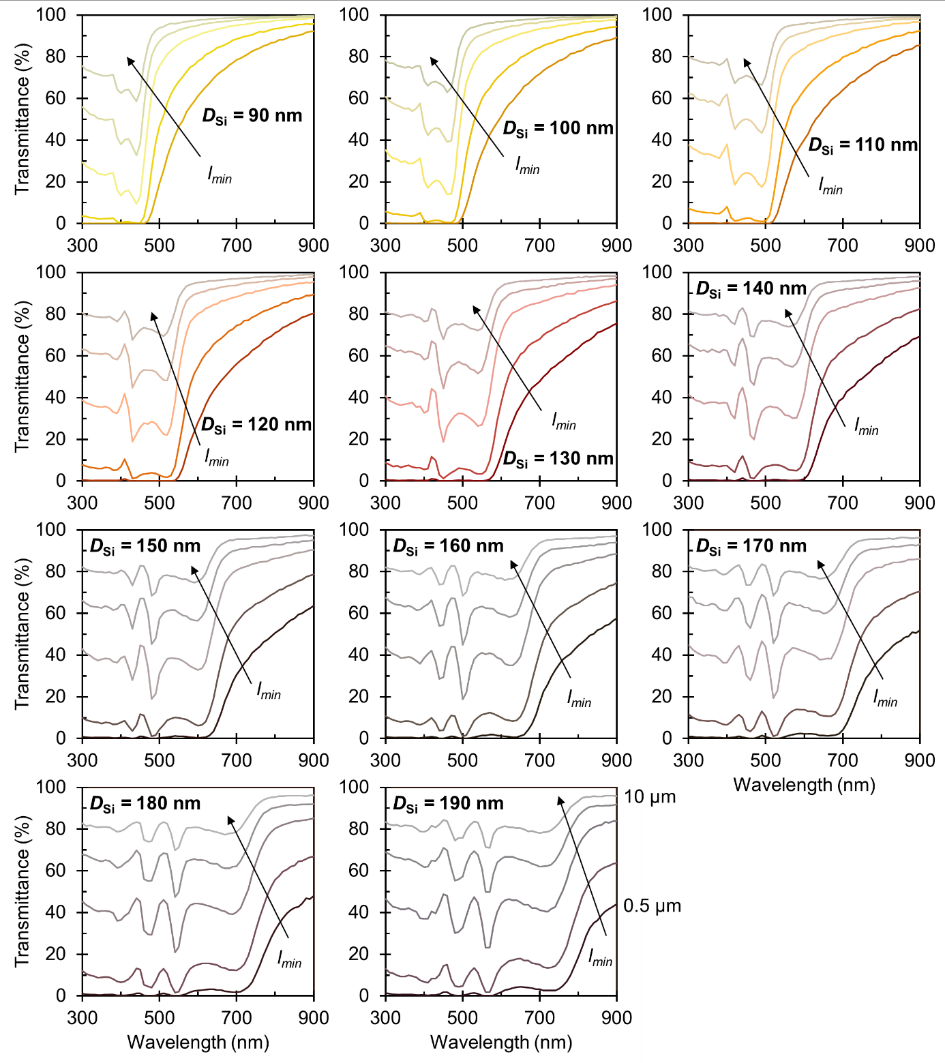


Figure S5. Simulated transmittance spectra of Si NP-dispersed resin films with Si NP diameters ranging from 90 nm to 190 nm. The minimum mean free paths (*l_min_*) of photons were set to 0.5, 1.0, 2.5, 5.0, and 10 µm. The color palettes representing transmission colors shown in Figure 3b of the main text were derived from these spectra.


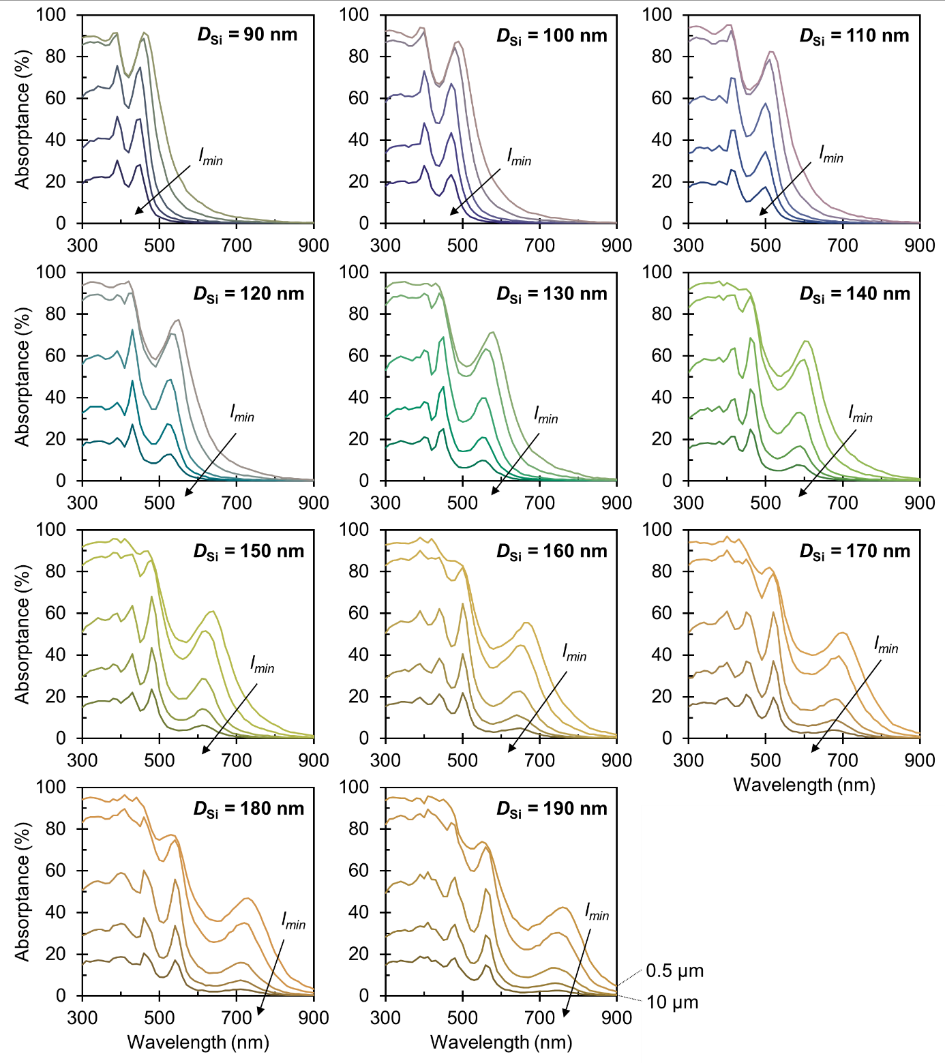


Figure S6. Simulated absorptance spectra of Si NP-dispersed resin films with Si NP diameters ranging from 90 nm to 190 nm. The minimum mean free paths (*l_min_*) of photons were set to 0.5, 1.0, 2.5, 5.0, and 10 µm.


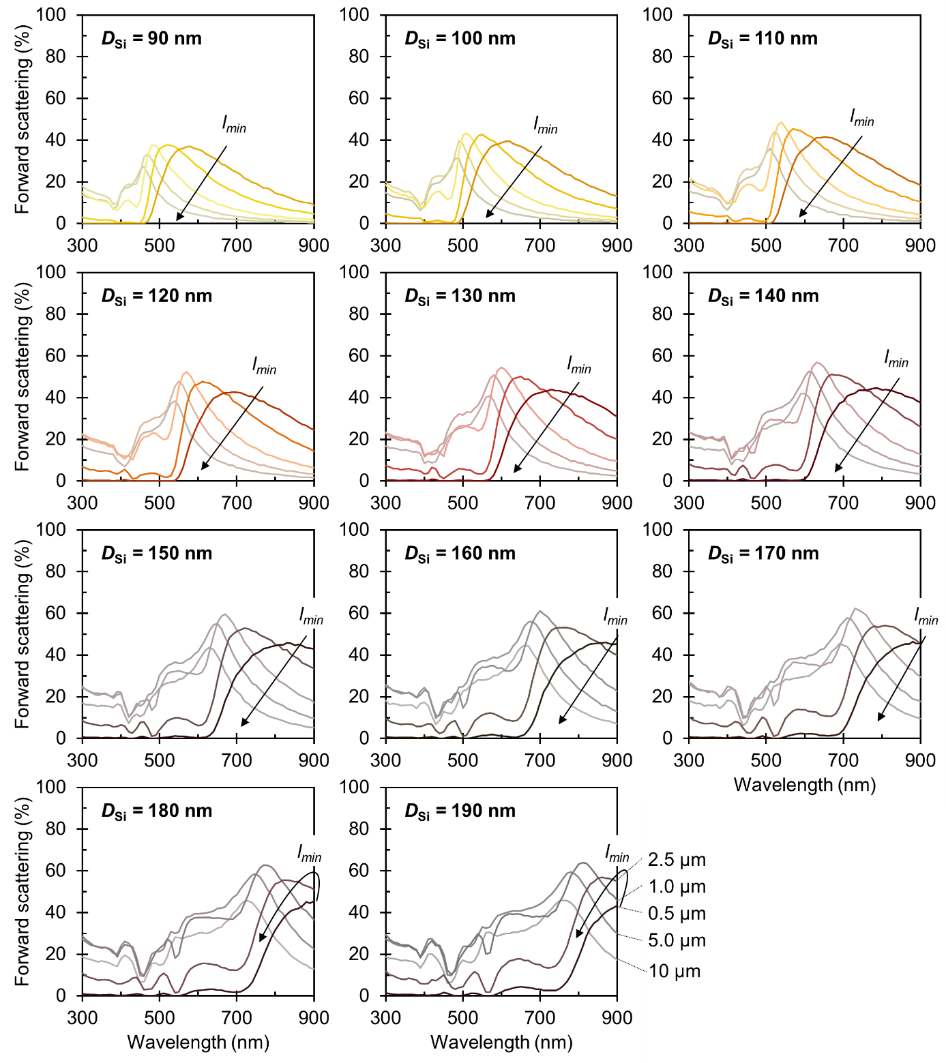


Figure S7. Simulated forward scattering spectra of Si NP-dispersed resin films with Si NP diameters ranging from 90 nm to 190 nm. The minimum mean free paths (*l_min_*) of photons were set to 0.5, 1.0, 2.5, 5.0, and 10 µm.


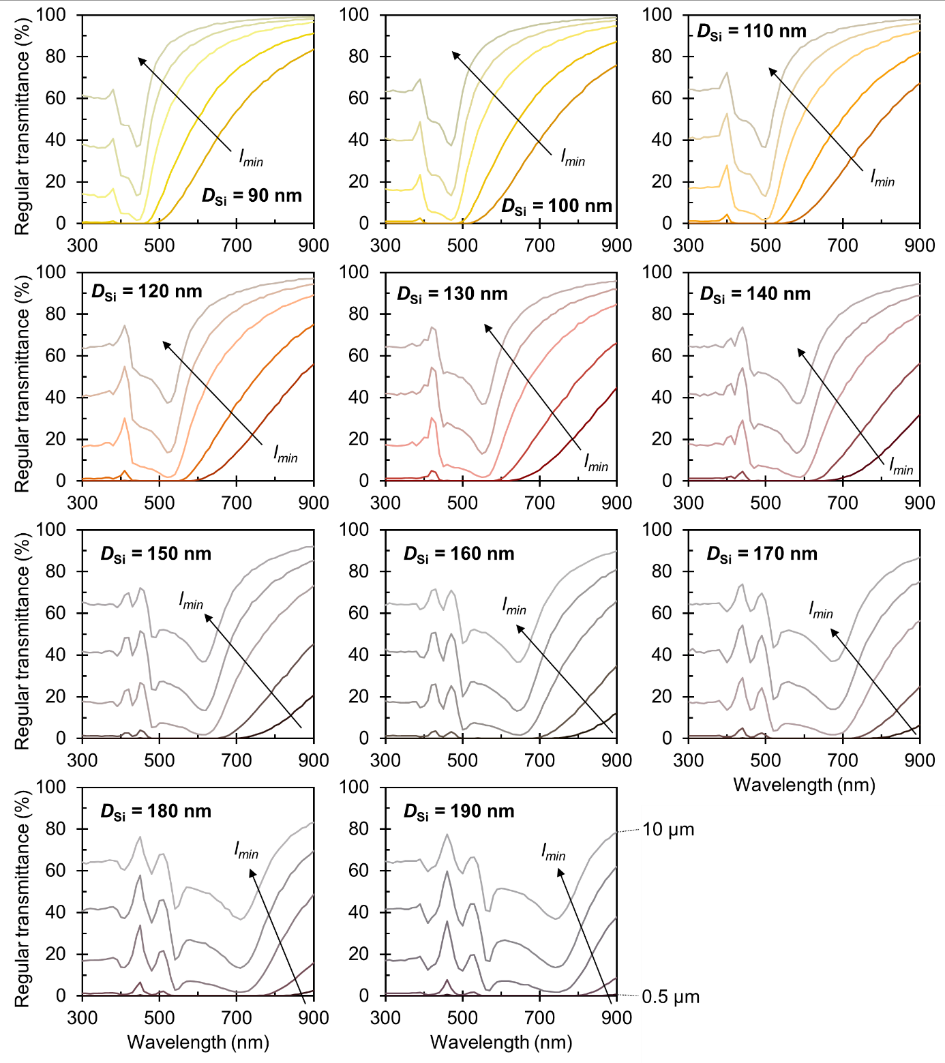


Figure S8. Simulated regular transmittance spectra of Si NP-dispersed resin films with Si NP diameters ranging from 90 nm to 190 nm. The minimum mean free paths (*l_min_*) of photons were set to 0.5, 1.0, 2.5, 5.0, and 10 µm.


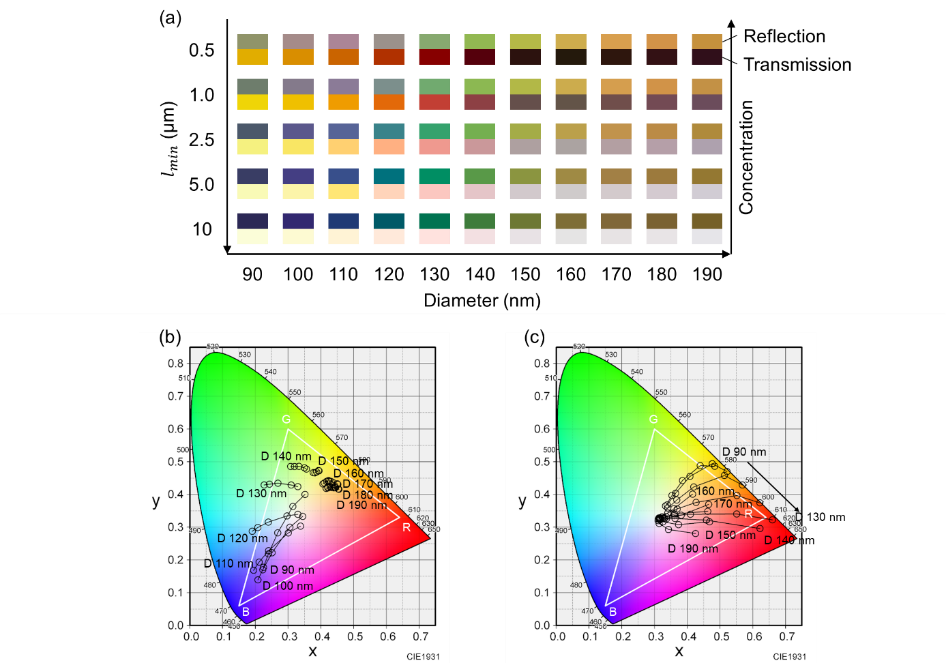


Figure S9. (a) Simulated sRGB color palettes for reflection (upper row) and transmission (lower row), derived from the reflectance and transmittance spectra shown in Figure S4 and S5. (b, c) CIE 1931 chromaticity diagrams plotting the reflection (b) and transmission (c) colors shown in (a). The white triangles indicate the color gamut of sRGB.

**5. Estimation of average diameter, size distribution, and concentration of size-separated Si NP dispersions, and the effect of size distribution on the scattering color**

The particle characteristics of Si NP dispersions were estimated by fitting the calculated extinction spectra to measured spectra, assuming a Gaussian size distribution.^[1,2]^ The extinction (absorbance) of Si NPs dispersed in methanol was measured using a microvolume spectrometer (NanoPhotometer NP80, Implen). Calculated extinction spectra were derived from Mie theory, incorporating a Gaussian size distribution. The measured and calculated spectra were differentiated and then fitted using the average diameter (*D*_ave_) and standard deviation (*σ*) of the distribution as fitting parameters. To compare the size distribution widths across different Si NP dispersions, we used the coefficient of variation (CV = *σ* / *D_ave_*) as a metric. The concentration of Si NPs was determined from the measured extinction intensity using the Beer-Lambert law. Figure S10 shows the measured and calculated extinction spectra for a subset of the dispersions shown in Figure 4c. The calculated spectra closely reproduced the measured ones, confirming the accuracy of this fitting process and the extracted parameters.


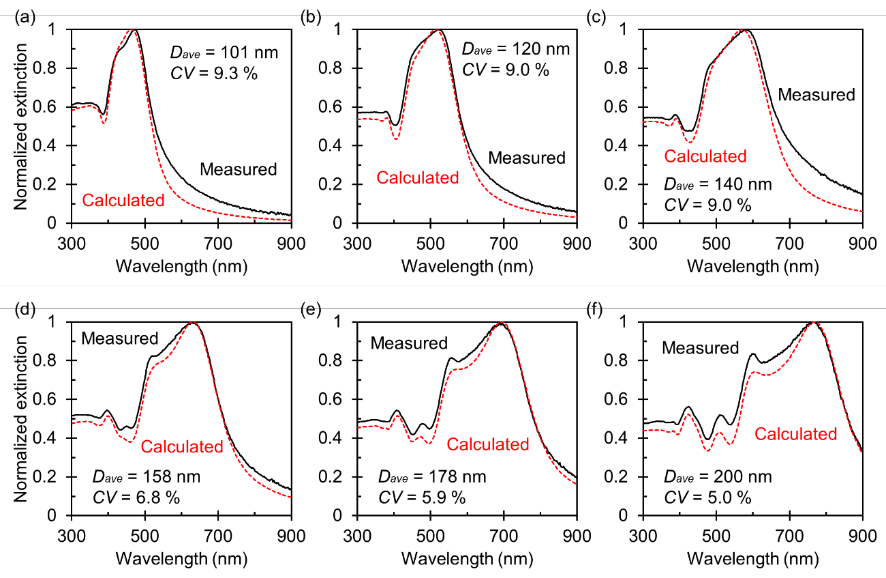


Figure S10. Normalized measured (black curves) and calculated (red curves) extinction spectra of Si NP dispersions. The estimated average diameter (*D*_ave_) and coefficient of variation (CV) of the size distribution are indicated in each graph.


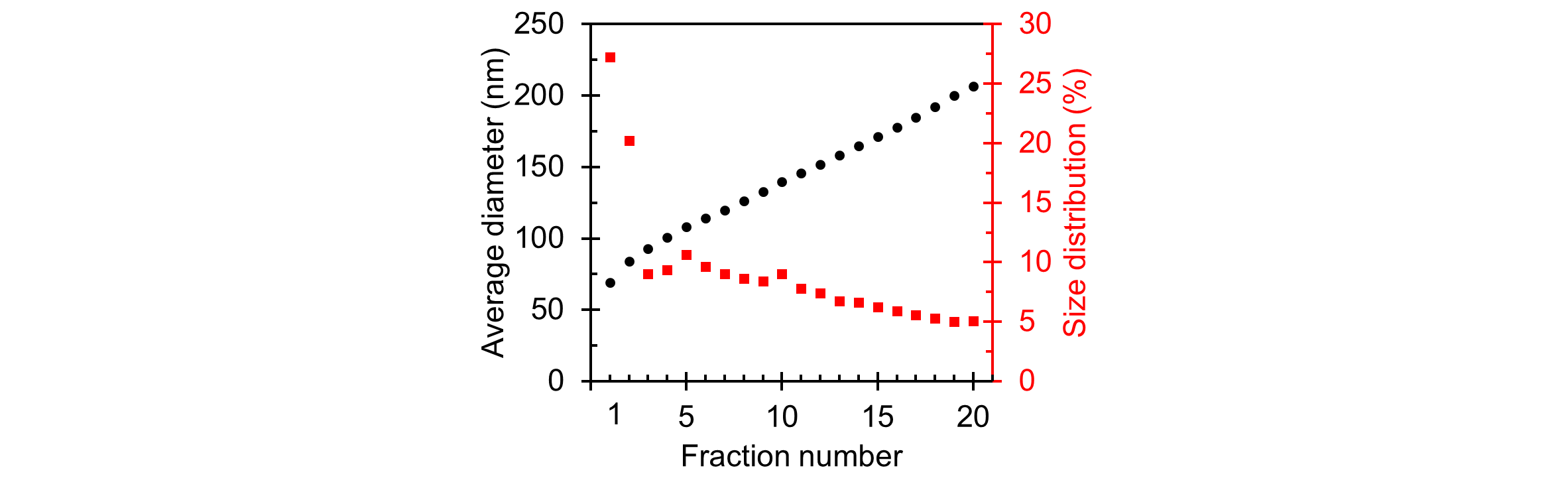


Figure S11. Estimated average diameters (*D*_ave_, black dots) and size distribution parameters (CV, red squares) of the Si NP suspensions shown in Figure 4c of the main text. These values were obtained by fitting the extinction spectra presented in Figure 4d of the main text.


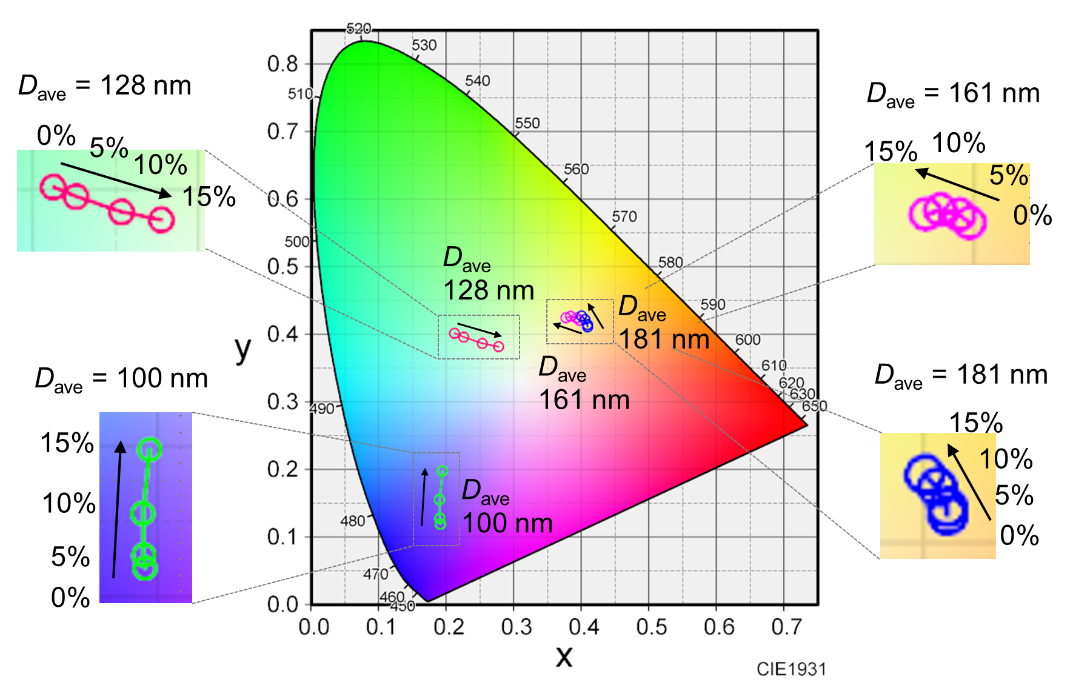


**Figure S12.** CIE chromaticity diagram derived from the calculated backward scattering spectra of Si NPs in a resin matrix (*n* = 1.49). The average diameters (*D*_ave_) were set to 100, 128, 161, and 181 nm, with the size distributions (CV) of 0, 5, 10, and 15%. The arrows indicate the direction of increasing size distribution width.

**6. Effect of SiO_2_ shell on the scattering properties of Si NPs**

As shown in Figure S13, printed bare Si NPs (without shells) undergo aggregation during the solvent evaporation process, which degrades color saturation. To maintain sufficient inter-particle spacing, the NPs were coated with amorphous SiO_2_ shells via the Stöber method. The shell thickness was controllable by adjusting the amount of TEOS and/or catalysts. Since the refractive index of the SiO_2_ shell (*n* ~1.46) is very close to that of the resin (*n* ~1.49), the shell remains effectively “invisible” to the incident light. Indeed, as shown in Figure S14a, the calculated scattering spectra of a Si NP coated with a SiO_2_ shell (*n* = 1.46) and one embedded directly in a resin matrix (*n* = 1.49) are almost identical, regardless of the shell thickness. Furthermore, the optical response is insensitive to small variations in the shell refractive index (Figure S14b). The data in Figures 5–7 in the main text confirm that the shells effectively prevent near-field coupling. Notably, the increase in NP size due to shell formation does not affect the ink rheology, given the low volume fraction of Si@SiO_2_ NPs in inks (< 0.3 vol.%).


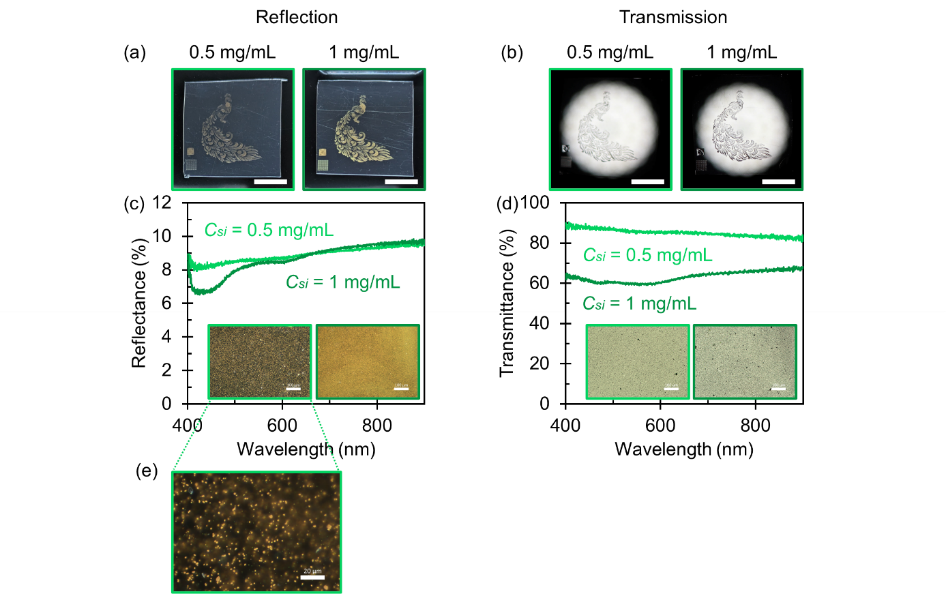


**Figure S13.** (a, b) Reflection (a) and transmission (b) images of inkjet-printed resin films containing bare Si NPs (without shells). The average diameter of the Si NPs was 133 nm, with concentrations of 0.5 mg/mL (left) and 1 mg/mL (right). Scale bars: 1 cm. (c, d) Measured reflectance (c) and transmittance (d) spectra of the inkjet-printed films. The insets display dark-field optical microscope images of the films. Si NP concentrations are 0.5 mg/mL (left) and 1 mg/mL (right). Scale bars: 100 µm. (e) High-magnification optical microscope image corresponding to panel (c). Aggregated Si NPs exhibit a yellowish color, whereas isolated Si NPs of this diameter appear green. Scale bar: 20 µm.


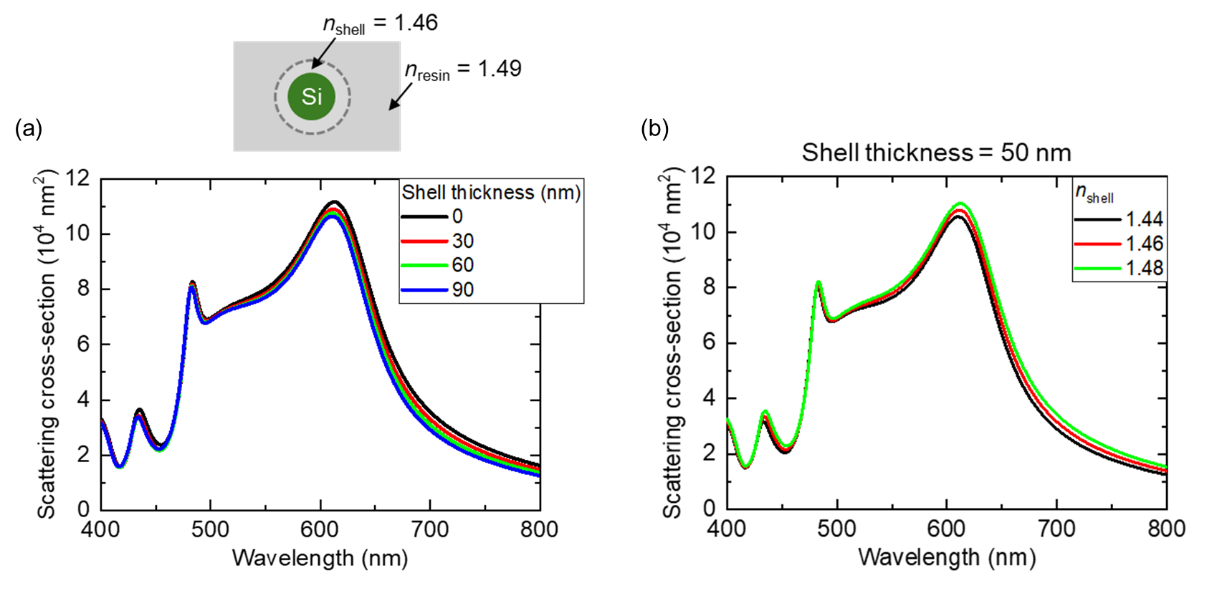


**Figure S14.** (a) Calculated scattering spectra of a single Si NP with varying SiO_2_ shell thicknesses (0 to 90 nm) embedded in a resin matrix (*n*_resin_ = 1.49). (b) Calculated scattering spectra of a single Si NP with 50 nm-thick shells having slightly different refractive indices (*n*_shell_ = 1.44, 1.46, 1.48) embedded in a resin matrix (*n*_resin_ = 1.49). The Si core diameters are fixed at 150 nm.

**7. Optimization of ink composition**

Inkjet printing inks typically consist of a pigment, a binder, and a solvent. In our formulation, Si NPs served as the pigment, an acrylic resin as the binder, and water as the solvent. To optimize the ink properties, we first prepared resin-only inks (without Si NPs) to determine the resin concentration that yielded a suitable viscosity for inkjet printing. For stable and reproducible inkjet printing, the ink viscosity needed to fall within the operating specifications (5–40 mPa·s) of the inkjet printhead used in this work (Microjet IJHD-1000). As shown in Figure S15, the ink could be ejected from the printhead at resin concentrations ranging from 4 to 20 wt.%. As is evident from the printed dot images, higher resin concentrations led to superior dot morphology, characterized by higher circularity and better placement accuracy. However, high resin concentration promotes the agglomeration of Si NPs as well as the ink solvent evaporation at the nozzle orifice. This resulted in nozzle clogging during printing and, consequently, poor reproducibility. Based on these data, we set a resin concentration to 16 wt.%, which balances dot morphology quality and long-term ink stability. It should be noted that the Si NPs did not significantly affect the ink viscosity due to their very low concentration (< 0.3 vol.%).


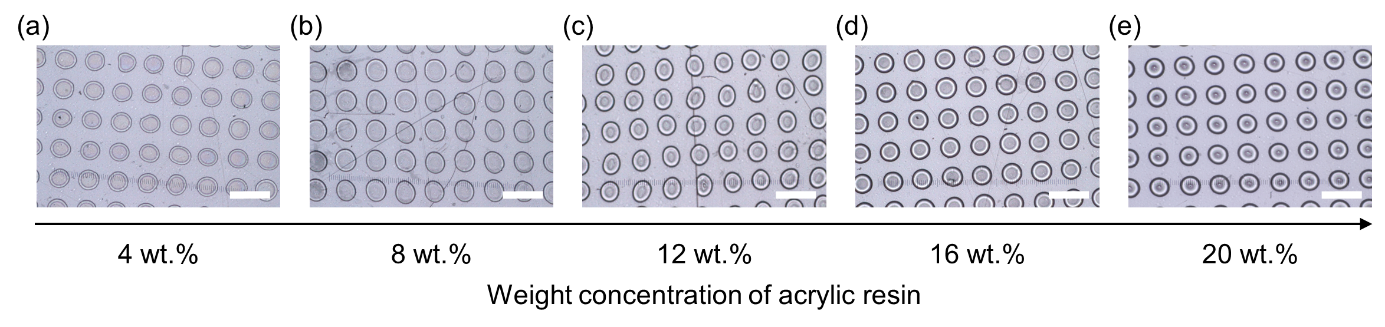


**Figure S15.** Bright-field optical microscope images of inkjet-printed acrylic resin. The printed inks consisted of acrylic resin and Milli-Q water at weight concentrations of 4 wt.% (a), 8 wt.% (b), 12 wt.% (c), 16 wt.% (d), and 20 wt.% (e). All images were captured after solvent evaporation using an upright reflected-light optical microscope (BX60M, Olympus). Scale bars: 400 µm.

**8. Optimization of inkjet parameters**

The inkjet parameters were optimized based on the manufacturer’s recommendations for stable operation. For the piezoelectric inkjet printhead used in this study (Microjet IJHD-1000), stable droplet flight is typically achieved at velocities between 7 and 8 m/s. First, the ejection frequency was set to 120 Hz; this sufficiently low frequency avoids acoustic interference from preceding ejection pulses, which can destabilize ejection at higher frequency (> 1000 Hz). Next, the pulse width of the driving voltage was optimized to maximize the droplet velocity. Under this condition, the pressure wave generated by the piezoelectric device matches the acoustic resonance of the ink chamber, resulting in an optimal width of 60–70 μs. The driving voltage was then adjusted within the range of 25–30 V to maintain the droplet velocity at the target 7–8 m/s.

The substrate was maintained at room temperature (~25 °C) because substrate heating increases the risk of ink solidification and nozzle clogging due to accelerated solvent evaporation at the nozzle orifice. Furthermore, the evaporation rate of the solvent (water) is sufficiently high at room temperature to ensure proper ink drying after deposition.

Under these optimized conditions, highly circular dots could be printed stably and continuously onto a polyethylene terephthalate (PET) film. Figure S16 shows an example of the printed dot array using an ink consisting of Si NPs dispersed in the resin solution at the concentration of 2 mg/mL.


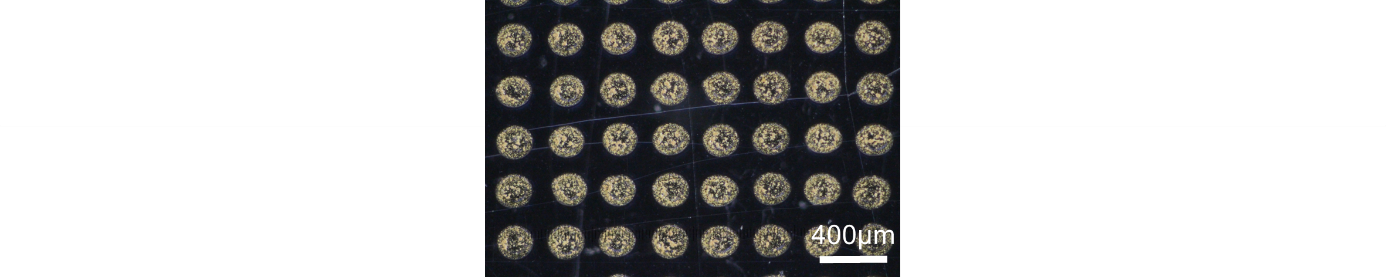


**Figure S16.** Dark-field optical microscope image of inkjet-printed dots. Si NP concentration was 2 mg/mL in the ink. The image was captured using an upright reflected-light optical microscope (Olympus BX60M). Scale bar: 400 µm.

**9. Reflection and transmission imaging and spectral measurement**


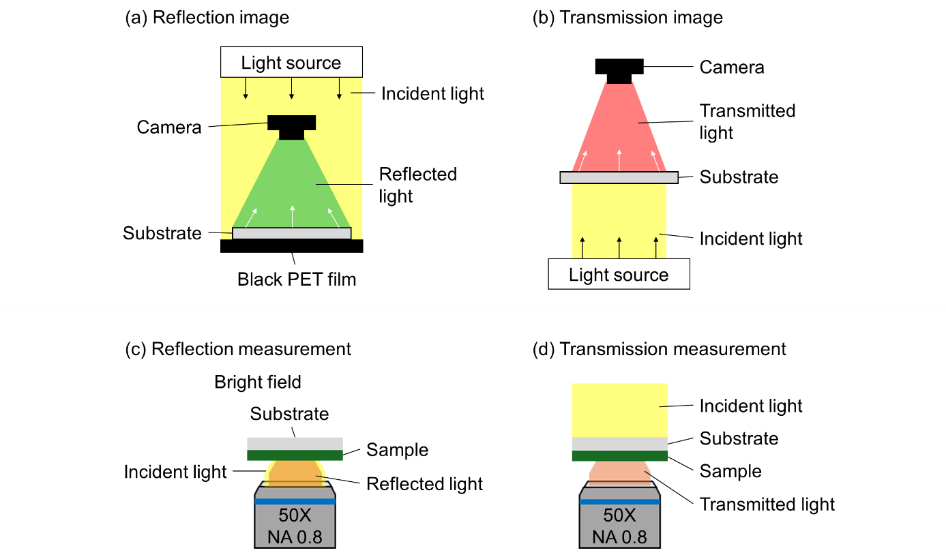


**Figure S17.** (a, b) Setups for capturing reflection (a) and transmission (b) images. Natural sunlight was used for reflection imaging, while an artificial solar light source (Seric SOLAX-iO, color temperature: 6500 K) was used for transmission imaging. Note that Figures 7p and 7q in the main text were captured under white fluorescent room lighting. (c, d) Schematics of the setups for reflection (c) and transmission (d) imaging and spectral measurements using the optical microscope (Nikon Ti-U).

**10. Dark-field optical microscope image of the peripheral region of a dot**

Due to the coffee-ring effect, the Si NPs were non-uniformly distributed within each printed dot: the NP concentration is higher in the peripheral region than that in center. However, the near-field coupling between NPs is effectively prevented by the SiO_2_ shells even in the peripheral region, and individual NPs can be resolved in the dark-field microscope images shown in Figure S18.

Generally, the coffee-ring effect can be mitigated by adding a co-solvent with a higher boiling point and lower surface tension than water.^[3,4]^ Such a mixed-solvent formulation induces an inward Marangoni flow, which counteracts the outward capillary flow during evaporation, thereby suppressing the coffee-ring effect. We plan to adopt this strategy in future work to achieve more uniform dot formation.


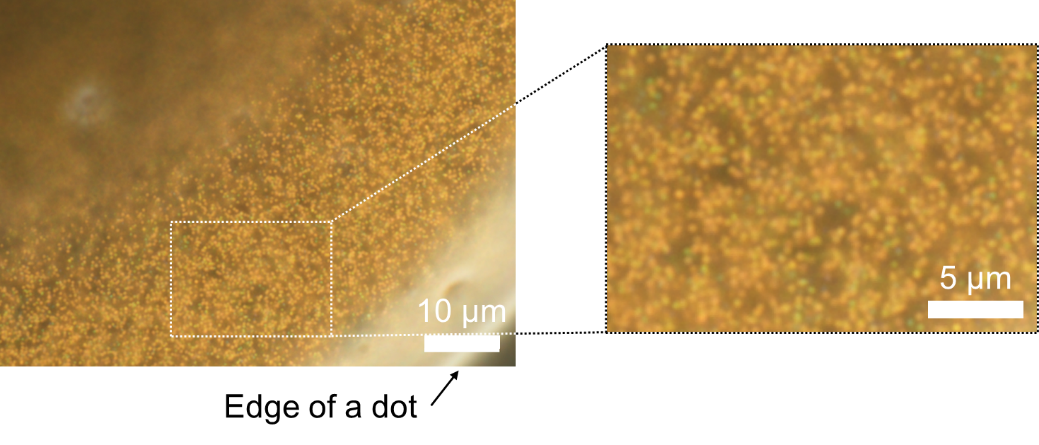


**Figure S18.** Dark-field optical microscope images of the peripheral region of the printed dot shown in Figure 5b of the main test. The right panel displays a magnified view. The Si core diameter is 181 nm.

**11. Digital designs with simulated reflection and transmission colors, and the procedure for printing a multicolor pattern**

Figure S19a displays the bitmap images corresponding to Figure 7a in the main text, colored based on the simulation results presented in Figure 3. The minimum mean free paths and Si NP diameters used in the simulations approximate the experimental conditions shown in Figure 7b-m. Good agreement is observed between the simulation and the experiment results.

Figure S19b illustrates the printing process of a multicolor peacock pattern using a single-head inkjet printer. First, a Si@SiO_2_ NP ink with a Si core diameter (*D*_Si_) of 128 nm was printed. Next, an ink with *D*_Si_ = 100 nm was printed in a different pattern on the same substrate, followed by inks with *D*_Si_ = 161 nm and, finally, *D*_Si_ = 181 nm in their respective patterns. The resulting pattern exhibited multiple printed colors. The patterns shown in Figure 7n-r were printed using this procedure.


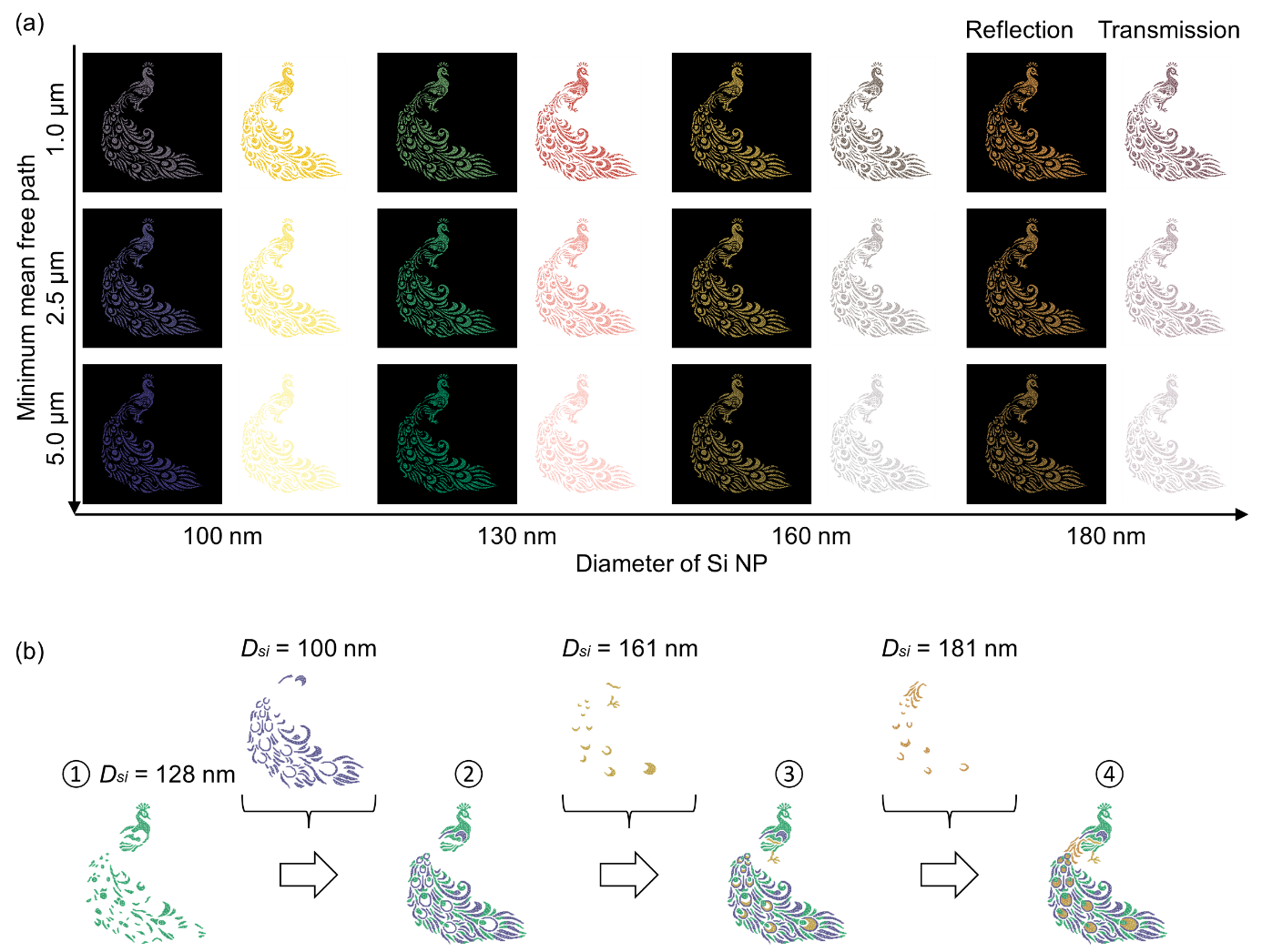


Figure S19. (a) Digitally designed bitmap images with simulated reflection and transmission colors. The simulation conditions approximate the experimental conditions shown in Figure 7b-m in the main text. (b) Schematic of the multicolor inkjet printing procedure. Inks containing Si NPs with different diameters were sequentially printed in distinct patterns on the same substrate.

**Reference**

1. Hinamoto, T., S. Hotta, H. Sugimoto, and M. Fujii, “Colloidal Solutions of Silicon Nanospheres toward All-Dielectric Optical Metafluids,” *Nano Letters* 20, no. 10 (2020): 7737–7743. https://doi.org/10.1021/acs.nanolett.0c03295.
2. Negoro, H., H. Sugimoto, and M. Fujii, “Helicity-Preserving Optical Metafluids,” *Nano Letters* 23, no. 11 (2023): 5101–5107. https://doi.org/10.1021/acs.nanolett.3c01026.
3. Park, J., and J. Moon, “Control of Colloidal Particle Deposit Patterns within Picoliter Droplets Ejected by Ink-Jet Printing,” *Langmuir* 22, no. 8 (2006): 3506-3513. https://doi.org/10.1021/la053450j.
4. Nam, H., K. Song, D. Ha, and T. Kim, “Inkjet Printing Based Mono-layered Photonic Crystal Patterning for Anti-counterfeiting Structural Colors,” *Scientific Reports* 6, no. 1 (2016): 30885. https://doi.org/10.1038/srep30885.
